# Effects of Ethnic Attributes on the Quality of Family Planning Services in Lima, Peru: A Randomized Crossover Trial

**DOI:** 10.1371/journal.pone.0115274

**Published:** 2015-02-11

**Authors:** Maria-Elena Planas, Patricia J. García, Monserrat Bustelo, Cesar P. Carcamo, Sebastian Martinez, Hugo Nopo, Julio Rodriguez, Maria-Fernanda Merino, Andrew Morrison

**Affiliations:** 1 Interculturality and Gender Unit, School of Public Health and Administration, Universidad Peruana Cayetano Heredia, Lima, Peru; 2 Department of Public Health and Primary Care, Leiden University Medical Centre, Leiden, The Netherlands; 3 Epidemiology, STD and HIV Unit, School of Public Health and Administration, Universidad Peruana Cayetano Heredia, Lima, Peru; 4 Gender and Diversity Division, Inter- American Development Bank, Washington, DC, United States of America; 5 Office of Strategic Planning and Development Effectiveness, Inter- American Development Bank, Washington, DC, United States of America; 6 Education Division, Inter- American Development Bank, Washington, DC, United States of America; 7 Executive Vice-President Division, Inter- American Development Bank, Washington, DC, United States of America; NHS lothian and University of Edinburgh, UNITED KINGDOM

## Abstract

Most studies reporting ethnic disparities in the quality of healthcare come from developed countries and rely on observational methods. We conducted the first experimental study to evaluate whether health providers in Peru provide differential quality of care for family planning services, based on the indigenous or mestizo (mixed ethnoracial ancestry) profile of the patient. In a crossover randomized controlled trial conducted in 2012, a sample of 351 out of the 408 public health establishments in Metropolitan Lima, Peru were randomly assigned to receive unannounced simulated patients enacting indigenous and mestizo profiles (sequence-1) or mestizo and then indigenous profiles (sequence-2), with a five week wash-out period. Both ethnic profiles used the same scripted scenario for seeking contraceptive advice but had distinctive cultural attributes such as clothing, styling of hair, make-up, accessories, posture and patterns of movement and speech. Our primary outcome measure of quality of care is the proportion of technical tasks performed by providers, as established by Peruvian family planning clinical guidelines. Providers and data analysts were kept blinded to the allocation. We found a non-significant mean difference of -0·7% (p = 0·23) between ethnic profiles in the percentage of technical tasks performed by providers. However we report large deficiencies in the compliance with quality standards of care for both profiles. Differential provider behaviour based on the patient's ethnic profiles compared in the study did not contribute to deficiencies in family planning outcomes observed. The study highlights the need to explore other determinants for poor compliance with quality standards, including demand and supply side factors, and calls for interventions to improve the quality of care for family planning services in Metropolitan Lima.

## Introduction

Ethnic inequalities in health outcomes, access to and utilization of healthcare have been reported in Peru.[1,2] However there are very few studies examining how ethnicity affects the quality of healthcare services received by patients. While observational studies have collected patients’ perceptions of ethnic discrimination in healthcare services,[3,4] to date no experimental data has been gathered on the mechanisms that may drive such perceptions. Furthermore, international literature has reported how health providers contribute to creating ethnic disparities across a wide range of clinical services,[5,6] including family planning (FP),[7] however there is little evidence in the context of a developing country such as Peru.

Through the provision of contraceptive information and services, FP allows individuals and couples to anticipate and attain their desired number of children and the spacing and timing of their births. There is evidence, mostly in the United States, indicating that contraception service providers make different recommendations according to ethnicity/race, with ethnic minorities more likely to report being pressured to limit their family size, and to receive counseling about sterilization and other highly effective contraceptive methods.[7–9] There is also a growing body of literature which suggests that even when providers report egalitarian beliefs and no intention to discriminate, it is possible to observe bias, discrimination or prejudice regarding ethnicity/race.[10] If FP providers systematically discriminate based on the ethnicity of a patient, they may contribute to ethnic disparities in the unmet need for contraceptive methods and in the disadvantage experienced when women are unable to control their fertility as desired.[11,12]

In Peru, after years of contradictory policy approaches in which health providers were either discouraged from delivering modern contraceptives or pressured to perform non-voluntary sterilizations, particularly to poor and indigenous women,[13] the Ministry of Health (MoH) issued clinical guidelines for FP in 2004 endorsing reproductive and sexual rights, as well as gender and diversity equality.[14–16] There are nevertheless continued ethnic disparities between indigenous and non-indigenous women in unmet needs for contraceptive methods (9.4% and 6.5%, respectively), and in use of modern contraceptives (21.9% and 34.7%, respectively).[17] It is unclear, however, whether these ethnic differences could be explained by women’s cultural preferences and behaviors, healthcare system factors, or service provider behavior.[7] This study aims to evaluate whether health providers in Peru might be contributing to ethnic disparities in the provision of FP services, by comparing providers’ adherence to national FP guidelines.

To evaluate guideline tasks performed by health providers during consultation, this study uses simulated patients (SPs), that is, individuals carefully recruited and trained to act as real patients. Our study is the first randomized controlled trial using SPs in face-to face interactions that managed to circumvent potential biases arising from unobserved heterogeneity. In the tradition of conventional audit tests in labor economics,[18] these studies have evaluated ethnic or racial disparities assigning auditors to pairs (e.g. one of each ethnicity or race), and matching them in equivalent characteristics. Such a design makes it difficult to assure that other unobserved idiosyncratic characteristics beside ethnicity are not affecting outcomes.[19] Recent audit studies addressed this limitation by randomizing ethnic/racial attributes within each quality pair in tests using photographs and surnames sent in résumés as ethnoracial proxies,[6] and in Implicit Association Tests,[20] but to our knowledge this is the first time it is implemented using a SP technique that assesses face-to-face interactions with health providers. Furthermore, the study is the first of its kind in experimentally testing how health providers might contribute to creating ethnic disparities in healthcare in developing countries.

## Materials and Methods

### Study design and participants

We undertook a two period, crossover randomized controlled trial, with 1:1 allocation ratio to evaluate whether patients’ ethnic characteristics trigger differences in the quality of FP services in Metropolitan Lima, the capital of Peru. Lima is a melting pot of ethnoracial populations, representing the rich diversity of the country, where 51% of the population self identifies as mestizo (mixed ethnoracial ancestry), 34% indigenous Quechua, 5% indigenous Aymara, 4% Amazonian ethnic groups, 3% white, 1% black and 2% other.

**Fig 1:**
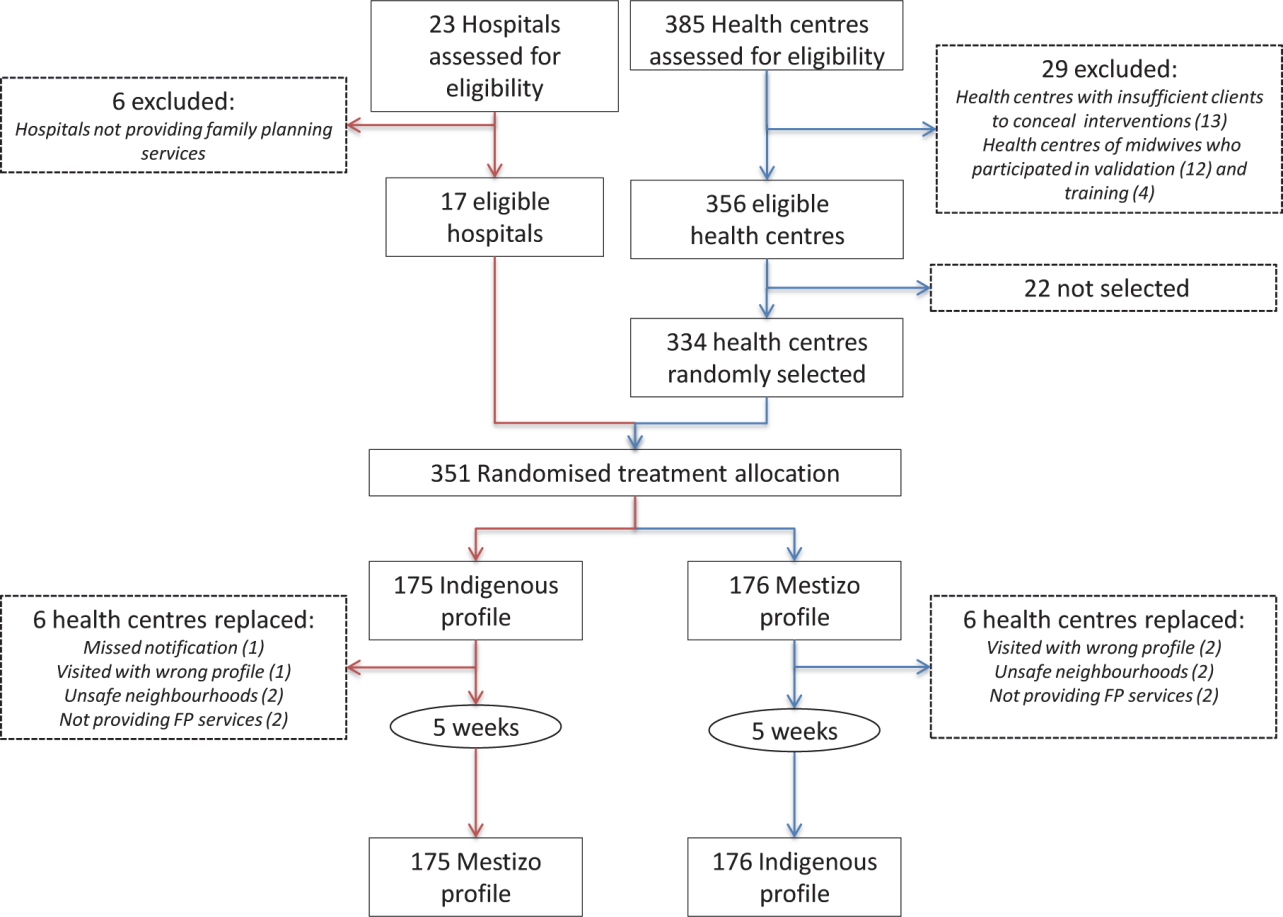
Trial profile.

The study was conducted during September 5 and November 27, 2012 in a sample of 351 health establishments operated by the MoH, which offer FP services provided by midwives. From a total of 408 MoH establishments in Lima, we excluded six specialized hospitals that did not offer FP services, 13 small health posts which did not provide care to a sufficiently large number of patients to enable the SP to pass undetected, and 16 health centres where we recruited midwives to assist in the study’s validation procedures and training. The eligible MoH establishments participating in the study are therefore 17 non-specialized hospitals and 334 health centers (Fig. 1).

The intervention consisted of unannounced SP visiting health establishments as indigenous Quechua and mestizo clients, seeking contraceptive advice following a standardized script. The study protocol was approved on September 5, 2012 by the Institutional Review Board (IRB) at Universidad Peruana Cayetano Heredia. The protocol for this trial and supporting CONSORT checklist are available as supporting information (see S1 Protocol and S1 CONSORT Checklist). The IRB exempted midwives from informed consent given that the study involved no more than minimal risk, did not adversely affect their rights and welfare, and could not practically be carried out without the waiver.[21] The protocol was published,[22] and registered with ClinicalTrials.gov (NCT01885858). Since the researchers initially involved in the study were economists and anthropologists, and within their disciplinary fields it is neither mandatory nor a common practice to register trials, we registered the study trial on June 15, 2013 after being informed of the specific standards for clinical trials and the related journal’s editorial policies in the medical field. The authors confirm that all ongoing and related trials for this intervention are registered.

**Fig 2:**
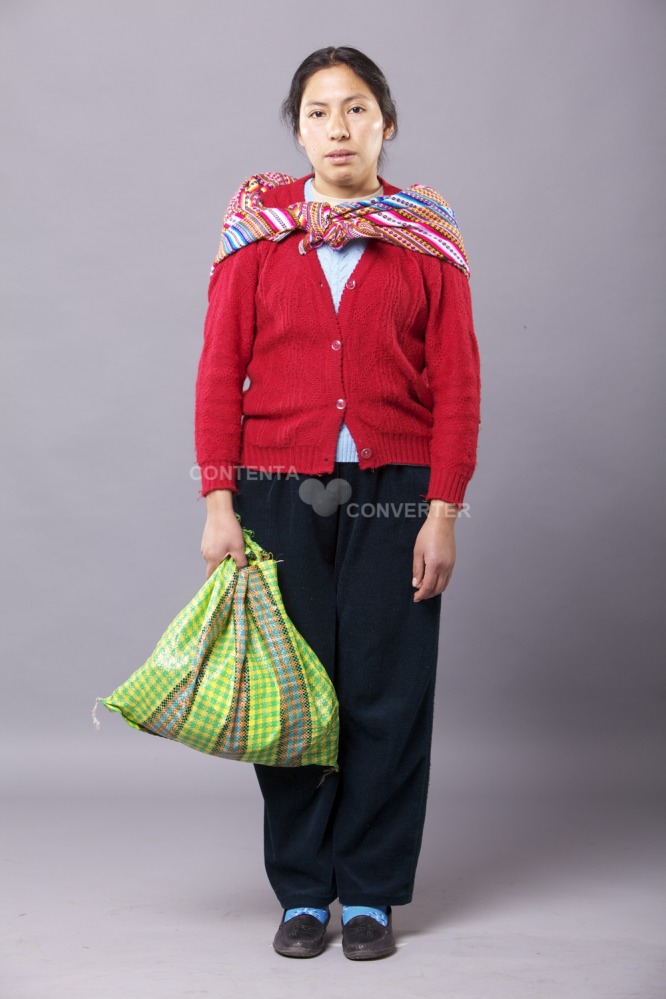
Indigenous profile. The indigenous profile wore long braids, no make-up, an *lliclla* (a traditional woven cloth that resembles a shawl), a woven cardigan, loose pants, her patterns of speech were slower, and her posture and movement more rigid than the mestizo profile. The individual in this manuscript has given written informed consent (as outlined in PLOS consent form) to publish her photograph.

**Fig 3:**
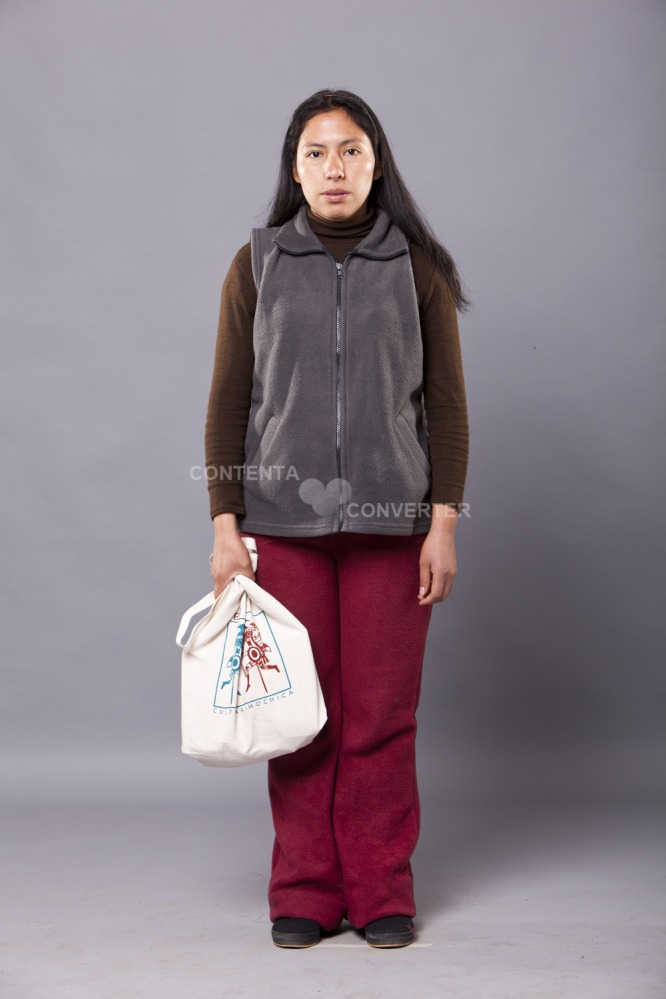
Mestizo profile. The mestizo profile wore a pony tail or loose hair, make-up, a vest made of polar fleece, tight pants, and culturally salient accessories, her patterns of speech were faster, and her posture and movement less rigid than the indigenous profile. The individual in this manuscript has given written informed consent (as outlined in PLOS consent form) to publish her photograph.

**Ethnic profiles.** The distinctive cultural attributes of ethnic profiles manipulated in the experiment were defined after a qualitative study, further adjusted during training, and later validated in a pilot study and a post-trial survey with a representative sample of midwives working in MoH FP services in Lima (see Planas *et al*.[22]). Since ethnicity is a multidimensional construct and its validity is context-driven,[23,24] we relied on midwives perceptions to identify the salient attributes they use to distinguish indigenous from mestizo clients. These attributes were culturally appropriate clothing, styling of hair, make-up, accessories, posture, and patterns of movement and speech. Accent was perceived as a salient attribute but we did not manipulate it since it was difficult to learn with realism and consistency within the timeframe of the study. Instead, we used Quechua accent as a criterion for recruiting the SPs. Given the interplay between racial and ethnic attributes,[25,26] we recruited SPs with phenotypic characteristics that made the distinctive cultural attributes relevant for midwives to distinguish indigenous from mestizo clients (see Planas *et al*.[22]). In order to manipulate ethnicity, while holding all other aspects of the patient constant (such as phenotype, physical attractiveness and attitudes), the same SP half the times sought FP services enacting an indigenous profile while the other half she did so enacting a mestizo profile (see Figs. 2 and 3).

The post-trial validation exercise confirmed that we successfully manipulated attributes that led local midwives to correctly infer the ethnic profile of the SP (S1 Table), that our indigenous and mestizo profiles were amongst the most common patient types visiting MoH FP services in Lima (S1 Fig.), and that extreme indigenous and lighter mestizo or white patients were rare (S2 Fig.).

**Script.** Based on the work by León *et al*.,[27] a script was developed for a new user of FP services interested in the possibility of using pills as her contraception method. During each FP visit SPs enacted a standardized script, introducing them as new user of FP services, low-income, uninsured, who did not complete secondary school, did not want more children for a few years (neither her nor her husband or partner), did not trust natural FP methods, and lacked experience with modern contraceptives. Her choice of method, if asked, was the pill. The only elements of the script that varied between ethnic profiles, if prompted, were information about birthplace and length of time living in Lima, since based on validation exercises long-time Lima residents preserving indigenous markers could be suspect. In addition, the script provided SPs excuses to avoid physical examination.

### Randomization, masking and blinding

We used a computer generated randomization schedule and permuted blocks of four (Microsoft Excel 2007) to assign health establishments to receive SPs enacting either indigenous and then mestizo profiles (sequence 1) or mestizo and then indigenous profiles (sequence 2) in two periods (Fig. 3). To help conceal the study, visits to the same establishment were conducted at least 5 weeks apart. A masked epidemiologist (CC) generated the allocation sequence, and while the data bank was open, only the investigator in charge of data collection (MEP) was aware of the assignment. Field supervisors and SPs were aware of the ethnic profile to be performed up to a week in advance. Because of the nature of the experiment, ethnic profiles had to be revealed to health providers. Yet since providers were unaware of the consultation’s purpose, they were effectively blinded to the experiment. Data analysis was also performed blind as to the arm allocation of the SP visits.

The order of health establishment visits was selected through probability multi-stage sampling. In the first stage, health centres were divided into three areas according to geographic proximity. In the second stage, the order of visits was randomized within each of the twelve healthcare networks in Lima. SPs were divided into three field teams, each led by one supervisor and assigned to one of the three areas with an ordered list of health establishments and a rotation period of six weeks. Turns were allocated systematically and groups of two establishments were assigned sequentially to SPs on the same team.

### Procedures

Of 132 female applicants recruited to act as SPs, ten aged between 25 and 35, with higher education degrees (most of them midwives) and with desirable phenotype, accent, cognitive and interpersonal skills successfully completed a two-week training program (see Planas *et al*.[22]). During the training we were assisted by an experienced actor and by 3 midwives who were recruited to act as supervisors and had previously received a two-day training on research ethics, project and monitoring procedures and data collection.

During fieldwork data was collected using Magpi, a mobile phone based application for data collection and transfer. Within the first 30 minutes after the FP consultation, SPs recorded whether or not the provider performed the guideline tasks on a checklist that required yes-no responses, as well as the cost and time spent to get the consultation.

Supervisors met with SPs before they visited the health establishments to verify correct characterization of the assigned ethnic profiles, and conducted daily exit-interviews to confirm that data had been correctly entered, to assess the SP’s judgment of provider performance, to detect potential departures from the script, and provide the SP with feedback. The exit-interview was audio taped and the supervisor sent daily notifications through her mobile confirming each supervision visit and whether the checklist was reported according to protocol. When an inconsistency was detected, it was corrected using printed supervision forms.

The database administrator corrected weekly data entry errors against the information collected through exit-interviews and supervision forms as well as other materials submitted daily by supervisors, such as payment bills, pills and prescriptions. The database administrator presented a weekly report of inconsistencies and incorrect data entries to the research team, which were corrected by checking with the fieldwork supervisor and/or SP. The research team met weekly with supervisors and the data manager to assess fieldwork progress and SP performance.

### Study endpoints

Considering that national guidelines for FP services are well known among providers, we compared provider’s adherence to these guidelines. The primary outcome of the study is the proportion of guideline technical tasks performed by health providers. This index of technical tasks includes items that relate to four competences: i) identify client’s needs; ii) respond to client’s needs by offering an appropriate range of contraceptive methods and explaining the characteristics of the method of choice; iii) verify client’s understanding of the characteristics of the method of choice; and iv) maintain rapport and schedule a follow-up visit.[16]. A detailed description of the 23 quality measurement tasks related to each of these technical competences has been published in Planas *et al*.[22].

Secondary outcome measures included: i) proportion of guideline socio-emotional tasks performed by providers, measured by tasks related to guideline skills necessary to establishing a friendly rapport with clients; ii) total length of visit; iii) total cost of the appointment, and; iv) total number of attempts from the first visit to the one when the FP consultation was completed (see Planas *et al*.[22]).

### Statistical analysis

The impact of the SP’s ethnic profile was estimated as the average difference in outcomes between the indigenous and mestizo profile visits in each center. We estimated a power over 99% to detect 10% differences in this index across 351 health establishments, using a standard deviation of 40, a level of statistical significance of 5% (two-sided). Linear regression analysis was used to control for time (day of the week, time of day), health establishment, and SP fixed effects to capture unobserved patient-specific heterogeneity (e.g. interviewing ability, physical attractiveness). A final specification includes a vector of provider and health establishment characteristics, such as age, gender, and ethnicity of providers, observed by the SP at the time of the visit. Our dataset is available as supporting information (see S1 Data).

## Results

Of the initial 351 health establishments selected, 12 had to be replaced because they did not offer FP services, were located in extremely unsafe neighborhoods, were visited with the wrong ethnic profile or notification of the checklist did not reach the server (Fig. 1). While health establishments were balanced by design, we could not guarantee that the same provider was observed during the two visits. However, based on provider characteristics collected during the interviews, it appears that providers did not vary systematically between SP profiles (S2–S3 Tables).

Table 1 reports the main results of the study. We find no statistically significant differences in the index of technical tasks, our primary measure of quality, based on the ethnic profile of patients. Results are similar in multivariable regression analysis (S4 Table). However, 37% compliance with standards of care is strikingly low for both ethnic profiles. Disaggregating the technical task quality index by competence in Table 2 shows that providers comply on average with only 14 percent of the tasks related with competence ii: respond to client’s need by offering the appropriate range of contraceptive options, and explaining characteristics of the method of choice. Average compliance with competencies i, iii, and iv ranges between 48 and 54 percent.

**Table 1:** Intra-clinics differences in index of technical tasks.

	Mestizo Profile	Indigenous Profile		
	mean (SD); n = 351	mean (SD); n = 351	Intra-Clinics Difference	p value
Compliance with quality of care guidelines (proportion of guidelines met)	36.2% (8.99%)	37.0% (9.01%)	-0.7	0.23

**Table 2:** Compliance with technical guideline competences.

Description of the competence	mean (SD); n = 702
Competence I: identify client's need considering her preferences and risk factors	53.4% (12.17%)
Competence II: respond to client's need by offering the appropriate range of contraceptive options and explaining characteristics of the method of choice (pill)	14.0% (5.81%)
Competence III: verify client's understanding of characteristics of method of choice (pill)	48.9% (50.02%)
Competence IV: maintain rapport and schedule a follow-up visit	50.9% (32.75%)

Results for secondary measures of quality tell a similar story. Table 3 shows no differences by ethnic profile on the quality index of socio-emotional tasks, though relative to technical tasks, providers score relatively high, performing on average 75 percent of the suggested guidelines. These results were confirmed in the multivariable regression analysis (S5 Table). Similarly, Table 4 shows no differences in the total cost and duration of the visit. On average, patients spend four Peruvian Soles ($1.40 US dollars) for a supposedly free consultation, and take 120 minutes from the moment they arrive at the establishment to the moment they leave the FP consultation. The FP consultation lasts on average 13 minutes, so 90% of time is spent waiting and on paperwork. Finally, SPs required on average 1.5 visits to the health establishment (on different days) before receiving a FP consultation. It is worth noting that this is the only statistic for which we find significant differences across ethnic profiles. Nonetheless, beyond statistical significance, the magnitude of such difference is too small (1.5 vs. 1.4 visits).

**Table 3:** Intra-clinics differences in index of socio-emotional tasks.

	Mestizo Profile	Indigenous Profile		
	mean (SD); n = 351	mean(SD); n = 351	Intra-Clinics Difference	p value
Compliance with quality of care guidelines (proportion of guidelines met)	75.0% (12.89%)	74.5% (12.42%)	0.5	0.59

**Table 4:** Intra-clinics differences in duration, cost of the visit, and number of visits necessary to receive FP services.

	Mestizo Profile	Indigenous Profile		
	mean (SD); n = 351	mean (SD); n = 351	Intra-Clinics Difference	p value
1. Total amount paid for the consultation (soles)	4.0 (2.55)	3.9 (2.50)	0.1	0.42
2. Total time in minutes, from reaching establishment until leaving consultation	119.8 (68.87)	114.5 (65.48)	5.1	0.22
2.1 Time in minutes, from reaching the establishment until it reaches the waiting room	58.2 (47.47)	54.4 (42.39)	3.0	0.34
2.2 Time in minutes, from entering the waiting room until it comes to consultation	49.2 (46.77)	49.2 (46.38)	0.2	0.95
2.3 Time in minutes, from entering consultation until leaves consultation	13.4 (12.56)	12.5 (10.33)	0.9	0.29
3. Total number of visits necessary to receive FP services	1.5 (0.71)	1.4 (0.73)	0.1	0.08

## Discussion

We conducted the first randomized controlled SP study to assess disparities in the provision of care based on the ethnic profile of patients in Lima, Peru. In contrast with observational evidence of ethnoracial discrimination in FP services located in Andean regions of Peru,[3,4] we do not detect systematic differences in the quality of care received in Lima by the ethnic profiles manipulated in the experiment. However, we do find alarming low levels of quality standards overall. On average, only 37% of technical tasks required by Peruvian FP guidelines are conducted, and compliance with key competencies such as offering the appropriate range of methods and explaining the method of choice are even lower. Furthermore, we documented that fees are charged, averaging $1.4 dollars for supposedly free services. To the extent that indigenous women are poorer, or have different health service preferences, expectations and needs, these deficient practices may act as barriers to access.

This study’s methodological approach, with random assignment of ethnic profiles, overcomes several limitations of previous experimental studies manipulating ethnicity/race to assess treatment outcomes and provider's management strategies in health care settings. First, by sending SPs to request FP services alternating randomly between indigenous and mestizo profiles, but using the exact same script, the study design controls for potential confounders from unobserved heterogeneity among pairs in a non-experimental settings. To our knowledge this study is the first randomized controlled trial using a SP technique that assesses face-to-face interactions in clinical settings that managed to circumvent potential biases arising from unobserved heterogeneity.

Second, by implementing pre-trial and post-trial validation exercises, we assure that midwives providing FP services in Lima correctly interpreted the constructed ethnic profiles. Third, service providers were unaware of the purpose of the visit, thus avoiding the Hawthorne effect and other biases related to announcing the SPs visits that are also inherent in other data collection strategies, such as direct patient observations, provider vignettes or patient exit surveys, where the presence of an observer or interviewer allows providers to improve or modify their usual behavior. Finally, we implemented rigorous quality control procedures to standardize the enacting of ethnic profiles and in data collection. Our experiment is also unique in terms of external validity, including 702 observations covering over 85% of health establishments in Lima.

Nonetheless, our study design has several limitations that are worth noting. Controlling for Hawthorne effects and blinding service providers to the intended treatment reduces our ability to collect midwife characteristics and to know whether the same midwife provided services to both ethnic profiles. We did not attempt to identify health providers in order to guarantee no more than minimal risk to providers and as a condition for the IRB's approval of the waiver of consent. Despite this, our results show that observed characteristics in both groups were successfully balanced, suggesting the absence of systematic sorting of midwives within health establishments based on a patient’s ethnic profile.

Second, asking the same SP to alternate ethnic profiles limited us to a specific and relatively short range of the ethnoracial continuum of characteristics of women that frequently visit family planning services in Lima, as perceived by midwives (see S1–2 Fig.). It cannot be excluded that not having manipulated accent in the experiment might have moved the ethnic profiles closer to each other, even if SPs were trained to minimize the salience of the standardized accent by giving short answers and by answering only if the provider asked specific questions. Furthermore, conducting the experiment required salient and credible ethnic traits of frequent users of MoH FP services in Lima, which excluded extreme indigenous and white phenotypes. Thus, our ethnic profiles might have achieved different results had they been set further apart. That our SPs passed unnoticed during the 702 visits attests to the realism of the constructed profiles and of the SPs interpretation.

Third, we trained SPs to be verbally passive and only answer the provider when he/she asks a specific question, giving short answers, avoiding chitchat, complaints and confrontational attitudes at any service point/station. It cannot be excluded that this standardized passivity contributed to the low quality of care observed in the study.

Our findings suggest that while the quality of FP services does not differ systematically based on the ethnic profile of our SPs, interventions are needed to improve quality of care for all patients in Lima. We stress caution in extrapolating this study’s findings to the state of all ethnic disparities in FP services in Lima or other parts of the country, since we examined a specific range of the ethnoracial continuum of characteristics of women that frequently visit family planning services in Lima.

Significant debate has revolved around the question of whether ethnicity can be reliably measured given the variety of ethnic dimensions and the challenges posed by the fact that their validity is context-driven. In Peru ethnic categories are porous and the boundary between indigenous and mestizo is particularly unstable given the extended Lamarckian mutable environmental theories of heredity and the supposedly egalitarian promise of the ideology of mestizaje (racial and cultural mixture).[28,29] In this context, our study examined validly and reliably ethnic differences for a population of FP patients with low-income and education living in Lima. Further experimental research on the impact of ethnicity on the quality of care, manipulating alternative ethnic attributes, in different clinical settings and across other regions, is merited.

## Supporting Information

S1 CONSORT Checklist CONSORT 2010 checklist. (DOC)

S1 Data All data analyzed in this manuscript is provided in .xls format. (XLSX)

S1 Fig Validation exercise to assess how indigenous are the SPs as perceived by midwives. (DOCX)

S2 Fig Validation exercise to assess how often midwives provide FP services to women resembling our SPs. (DOCX)

S1 Protocol Study protocol approved by the Institutional Review Board at Universidad Peruana Cayetano Heredia (English and original Spanish versions). (DOCX)

S1 Table Validation exercise to assess whether midwives correctly inferred the ethnicity of the SPs. (DOCX)

S2 Table Health clinics descriptive statistics reported by SPs. (DOCX)

S3 Table Health provider descriptive statistics reported by SPs. (DOCX)

S4 Table Differences in the technical task index using multivariate analysis. (DOCX)

S5 Table Differences in socio-emotional task index using multivariate analysis. (DOCX)
