# Supplementary material for: Effects of Ethnic Attributes on the Quality of Family Planning Services in Lima, Peru: A Randomized Crossover Trial
Source: PLoS One. 2015 Feb 11;10(2):e0115274. doi: 10.1371/journal.pone.0115274 (PMC4324646; doi:10.1371/journal.pone.0115274)
Supplement: S1 Fig — (DOCX) [file pone.0115274.s003.docx]

**Figure S1.** **Validation exercise to assess how indigenous are the SPs as perceived by midwives.** A representative sample of MoH midwives assessed a repertoire of 8 full-body photographs which included two of our simulated patients (the ones with the lightest and darkest skin color), and six other ethnic profiles that represent the ethnic diversity in Metropolitan Lima. We asked midwives to assess how indigenous were each of the 8 women using an ordinal scale ranging from zero to 10, with 0 being nothing indigenous and 10 completely indigenous. The figure shows the mean values of perceived indigenousness for every ethnic profile (for further details on the design of the ex-post validation exercise see Planas *et al*.[1]).

| **Indigenous PS**  **Mestizo PS** |
| --- |


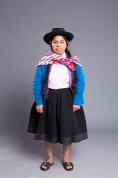

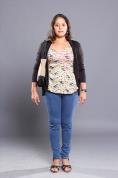

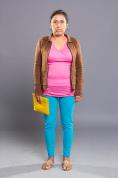

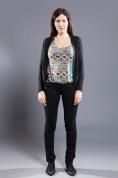

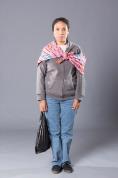

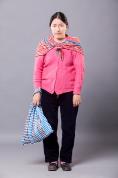

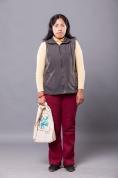

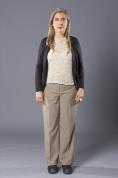


1. Planas ME, García PJ, Bustelo M, Carcamo CP, Ñopo HR, et al. (2014) Using standardized simulated patients to measure ethnic disparities in family planning services in Peru: Study protocol and pre-trial procedures of a crossover randomized trial. Washington, DC: Inter-American Development Bank. Available: http://publications.iadb.org/handle/11319/6387. Accessed 26 March 2014.
