# Supplementary material for: Effects of Ethnic Attributes on the Quality of Family Planning Services in Lima, Peru: A Randomized Crossover Trial
Source: PLoS One. 2015 Feb 11;10(2):e0115274. doi: 10.1371/journal.pone.0115274 (PMC4324646; doi:10.1371/journal.pone.0115274)
Supplement: S2 Fig — (DOCX) [file pone.0115274.s004.docx]

**Figure S2. Validation exercise to assess how often midwives provide FP services to women resembling our SPs.** A representative sample of MoH midwives evaluated a repertoire of 8 full-body photographs which included two of our SPs (the ones with the lightest and darkest skin color), and six other ethnic profiles that represent the ethnic diversity in Metropolitan Lima. The figure shows the percentage of midwives that described our SPs and the six other additional ethnic profiles as common clients (for further details on the design of the ex-post validation exercise see Planas et al.[1]).

| **Mestizo PS**  **Indigenous PS** |
| --- |


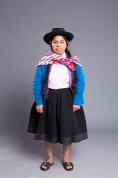

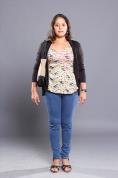

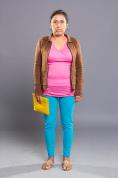

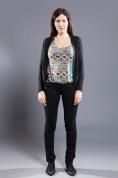

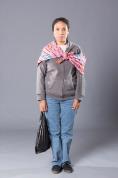

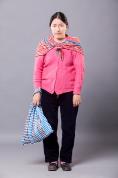

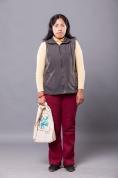

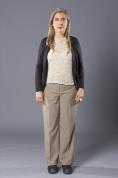


1. Planas ME, García PJ, Bustelo M, Carcamo CP, Ñopo HR, et al. (2014) Using standardized simulated patients to measure ethnic disparities in family planning services in Peru: Study protocol and pre-trial procedures of a crossover randomized trial. Washington, DC: Inter-American Development Bank. Available: http://publications.iadb.org/handle/11319/6387. Accessed 26 March 2014.
