# Supplementary material for: Effects of Ethnic Attributes on the Quality of Family Planning Services in Lima, Peru: A Randomized Crossover Trial
Source: PLoS One. 2015 Feb 11;10(2):e0115274. doi: 10.1371/journal.pone.0115274 (PMC4324646; doi:10.1371/journal.pone.0115274)
Supplement: S1 Protocol — (DOCX) [file pone.0115274.s005.docx]

**Protocol S1. Study protocol approved by the Institutional Review Board at Universidad Peruana Cayetano Heredia (English and original Spanish versions).**

**Quality of Health Services**

**In Indigenous Communities In Peru**

**Study Protocol**

**1. Problem and Conceptual Approach**

Like in other Latin American countries with similar histories of colonization, ethnoracial inequalities in access to health have been well documented Peru [[1-3](#_ENREF_1)]. Yet there is only limited evidence about ethnoracial disparities in the quality of health services. While existing qualitative studies and opinion polls provide information on perceptions of ethnoracial discrimination in health care services [[4-6](#_ENREF_4)], to date no quantitative data has been gathered on how a patient’s phenotype or ethnicity can influence the quality of health care received, and whether this contributes to ethnoracial disparities in the distribution of health, illness and well-being in the country [[2](#_ENREF_2), [7](#_ENREF_7), [8](#_ENREF_8)].

The present study evaluates whether in Peru the ethnoracial characteristics of patients determine disparities in the quality of health care received due to ethnoracial bias, prejudice or stereotypes held by the service provider; a scenario that has been reported extensively in international literature [[9](#_ENREF_9), [10](#_ENREF_10)]. This study adopts the term ‘ethnoracial’ given that in societies with unequal race relations, such as Peru, notions of ethnicity and race overlap analytically and in practice [[11-13](#_ENREF_11)]. Furthermore, this study defines race as a social, not biological, category that refers to different phenotypical characteristics (such as skin color) which gained significance in colonial contexts for distinguishing the European ‘us’ from the inferior ‘other’ [[11-17](#_ENREF_11)].

“The racial question in Peru”, indicates Twanama (1999), “conceals a mixture of variables against which people discriminate … the “choleo” (discrimination towards indigenous people) exercised against someone considered racially inferior due to his/her Indian background, is also evaluated according to socio-economic, educational and linguistic aspects, as well as migration status. It is not only that money or a university qualification that can ‘whiten’– to a certain degree – but that poverty or place of origin also indigenize” [[18:212](#_ENREF_18)]. As well as this multi-dimensionality, which characterizes ethnicity/race when it is intersected with other axes of social differentiation (such as class, gender and sexuality) [[4](#_ENREF_4), [18-20](#_ENREF_18)], discrimination in Peru is also contextual and situational. On the one hand, the locality or specific geographic space determines which ethnoracial characteristics and differences are salient, and who is recognized as indigenous, mestizo (individuals self-identified or perceived as having mixed ancestry) or white [[21](#_ENREF_21)]. On the other hand, the frequency of ethnoracial discrimination varies according to specific settings of social interaction (e.g. in schools, health services, when applying for a job.) [[4](#_ENREF_4)].

It is vital, therefore, to describe how discriminatory practices relate differently to diverse contexts and situations and to evaluate the specific and relative relevance of ethnicity/race as a motivation for discrimination, in order to help focus interventions aimed at reducing ethnoracial disparities in Peru. The present study intends to contribute to this goal by evaluating whether ethnoracial disparities exist in health care provision, and whether these disparities are determined by independent phenotypic characteristics or those associated with cultural traits.

With the aim of determining ethnoracial disparities in clinical care, this study will compare the degree of consistency in the clinical performance of health service providers when attending patients with diverse ethnoracial types. To do so, the study will measure providers’ compliance with clinical care standards related to: a) the duration and cost of the clinical consultation, and b) the quality of provider-patient communication during the clinical consultation, in terms of the socio-emotional aspects as well as related to the technical tasks, that is to say, recognition of the patient’s problem, diagnosis, treatment (such as advice, mediation, referral, supporting examinations) and follow-up. In addition, the quality of the patients’ itineraries will be evaluated during their visit to the health establishment, including interaction with specific ‘stations’ (for example, entrance/security, help desk or triage, appointment area, payment desk, doctor’s room).

**2. Objectives**

***General Objective***

Measure ethnoracial disparities in the quality of health services in Peru.

***Specific Objectives***

- Validate an ethnoracial typology that identifies indigenous and non-indigenous types to be used in the study.
- Compare the duration and cost of the clinical consultation between ethnoracially diverse patients.
- Compare the quality of provider-patient communication during the clinical consultation between ethnoracially diverse patients.
- Compare the quality of the itinerary during the visit to the health establishment between ethnoracially diverse patients.

**3. Methodology**

The present study consists of a randomized trial to compare discrepancies in the degree to which providers comply with health care quality standards when attending to ethnoracially diverse patients who visit family planning advice services provided by the Ministry of Health (MINSA). This clinical service provided by professional obstetricians has been selected because it has well-established protocols – including cultural competencies - approved by the MINSA, and because validated instruments and scripts for simulated patients are readily available [[22-24](#_ENREF_22)]. This selection is further justified by existing evidence in relation to ethnoracial disparities in the use of modern family planning methods [3]; a situation which could be caused due to characteristics of service provision (such as discrimination) or of demand (such as socio-cultural preferences). Specifically, this study aims to explore the causal relationship between ethnoracial characteristics of patients and the quality of provision of family planning advice.

Previous studies have evaluated disparities in the quality of service provision comparing health results between patients with different ethnoracial characteristics [4, 27-30]. One limitation of these studies, however, is that they do not control for the unobserved heterogeneity amongst patients. This, in turn, makes it difficult to evaluate whether other unobserved idiosyncratic characteristics, beyond ethnoracial characteristics, are affecting the results. To overcome this limitation, an experiment will be carried out inspired by a methodology used in labor economics known as ‘audit studies’, which aims to isolate the impact of ethnoracial and/or gender discrimination. For example, when evaluating a job applicant discrimination is understood to exist when two people with identical observable characteristics, such as level of education, place of residence, income and work experience, with the exception of their ethnoracial or gender characteristics, receive differential treatment. Following this line of investigation, the simulated patient technique will be used to experimentally manipulate the perception of ethnoracial characteristics in order to measure disparities in the provision of health services. In addition, information on the profile of service providers (such as sex, age and profession) and on the health establishments will be collected using publicly managed data bases and reports provided by the simulation patients.

***3.1 Simulated Patient (SP) Technique***

The simulated patients are trained actors who seek medical care following a pre-defined and standardized script. These actors report their observations about medical care received in an objective manner after their visit to a health establishment. The simulated patient technique, implemented successfully by the School of Public Health and Administration at the Universidad Peruana Cayetano Heredia (FASPA/UPCH) on various occasions [[6](#_ENREF_6), [25](#_ENREF_25), [26](#_ENREF_26)], reduces both Hawthorne effects and the variability between patients. This technique also has the advantage of being able to capture more critical and reliable observations during patient exit interviews. This is because by informing the simulated patients about quality standards related to the actions they are observing, they have higher expectations regarding satisfaction. Finally, this technique helps to standardize quality criteria between all simulated patients. Usually, these quality criteria can vary according to an individual’s idiosyncrasies and culture, and may differ from or completely ignore official guidelines for medical care.

In order to manipulate the ethnoracial perception of the patients, two ethnoracial types have been defined: Type-1: Indigenous (indigenous phenotype + indigenous clothing) and, Type-2: Non-Indigenous (mestizo phenotype + western clothing). The two ethnoracial types will be constructed by randomly modifying the physical appearance of the actors, using different make-up, hairstyles and clothing. Each simulation patient will attempt to receive family planning advice, sometimes interpreting Type-1: Indigenous and on other occasions Type-2: Non- Indigenous. This will enable the study to avoid the potential bias that can result from unobserved heterogeneity between actors (for example, all the physical characteristics and other non-physical attributes such as body language, attitudes and manner of speaking).

***3.2 Sample Size and Sample Selection***

Given that the health establishment represents the unit of analysis in this study, the data set has been calculated to include all of the Ministry of Health establishments in Metropolitan Lima and Callao, with the exception of specialized clinics and health posts which do not receive the number of patients for family planning services that are required to enable the simulated patients to pass through the system undetected.

After rigorous recruitment and training (see section 4), 18 simulated patients - all women between 25 and 40 years old - will visit family planning services in a sample of 351 health establishments. The sample size was calculated considering that 702 observations – 2 per establishment - would enable a significant difference of 10% with a power of 73% to be detected, which would be acceptable according to international literature [[10](#_ENREF_10)].

The sample will be multi-staged. All of the hospitals in Lima and Callao will be selected (with the exception of specialized clinics) and a sample of health establishments categorized as I-3 and I-4 will be chosen at random. Next, the order in which the two ethnoracial types will visit each health establishment will be assigned at random: Order1, if Type-1: Indigenous is first and Type-2: Non-Indigenous is second, and Order2 if Type-2: Non-Indigenous is first and TYPE-1: Indigenous is second. These orders will be randomly assigned to complete size-4 blocks, where 4 corresponds to the number of health establishments. Finally, the visiting time will be set as the earliest available appointment.

***3.3 Instruments***

To implement the simulated patient technique, the following instruments have been developed:

1. **A simulated patient script** (see Annex 1), which defines the motive for the consultation, medical history and biographical and behavioral characteristics (such as civil status, place of origin, type of employment, uninsured, degree of assertiveness etc.) that every simulated patient should interpret during her visit to the health establishment and during the medical consultation. An adaptation of a script elaborated by León et al. (2008) (see Annex 1), the script used in this study will be validated during the training (see section 4).
2. **A Checklist of service quality indicators** (see Annex 2). Based on current regulations around quality standards for family planning services, a selection of key service provider behaviors have been identified, to avoid problems related to remembering all possible behaviors. Likewise, questions have been scripted that require only simple responses, such as ‘yes’ and ‘no’ – in order to avoid subjective judgment. The selected indicators capture the following dimensions of quality to be assessed during the consultation: a) duration and cost of the consultation (see items I-VI and X of the instrument) and, b) the quality of provider-patient communication during the consultation, including technical tasks (such as types of method offered, side-effects of the pill, how the pill works, follow-up etc.) as well as communication and socio-emotional factors (social interchange or a positive or negative emotional connection, for example) (see items VII and VIII of the instrument). In addition, the check list will record the flow and type of treatment at different stations visited within a single establishment (see item IX of the instrument). This check list will be submitted via cellular telephone and validated by experts (see section 4)
3. **A Simulated Patient Exit Interview** (see Annex 3) will be used during daily supervisions to control the quality of fieldwork. Basic data will be reported via cellular telephone and audio recordings will be made of qualitative interviews to capture information on the station itinerary during each visit, identify any difficulties experienced by the simulated patient during the visit, collect her suggestions and provide her with timely feedback.
   1. ***Econometric Analysis Model***

The main objective of the study is to identify a causal relationship between the ethnoracial characteristics of an individual and the quality of health care received. With this aim, simulated patients will be sent to request medical assistance while randomly interpreting two different ethnoracial types.

The baseline specification that will be estimated is:

| (1) | $Y_{\mathrm{ijt}}=\lambda_{t}+\varphi_{j}+\mu_{i}+ \beta{indigenous\_ethnicity race/}_{\mathrm{ijt}}+X_{\mathrm{ijt}}^{'}\delta+\varepsilon_{\mathrm{ijt}}$ |
| --- | --- |

where $Y_{\mathrm{ijt}}$denotes the outcome of interest for simulated patient i in hospital/health center j at day of the week t. $\lambda_{t}$ denotes time fixed effects, $\varphi_{j}$ denotes hospital/health center fixed effects that control for time-invariant hospital/health center characteristics, and $\mu_{i}$are patient fixed effects that capture unobserved patient-specific heterogeneity (e.g. body language, speech patterns and attitudes), $X_{\mathrm{ijt}}$ is a vector of exogenous health practitioner and hospital/health center observable time-variant characteristics (e.g. age, gender and race of health practitioner), ${indigenous\_ethnicity race}_{\mathrm{ijt}}$is an indicator variable that takes the value of one if the simulated patient interpreted the “Indigenous” ethnicity/race, and $\varepsilon_{\mathrm{ijt}}$is a random, idiosyncratic error term. Standard errors are clustered at the hospital/health center level to allow for correlation across patients within a hospital/health center. The parameter of interest $\beta$measures the impact of ethnoracial characteristics on the quality of health care received.

**4. Procedures**

In order to carry out the study, four phases of work will be undertaken, as described in the following section.

**4.1 Preparation Phase**

During this phase, the following activities will be carried out:

- Presentation of the study to relevant agencies of the Ministry of Health and submission of the protocol to the Ethics Committee at the Universidad Peruana Cayetano Heredia
- By adapting the systematic data collection techniques proposed by Weller & Romney [[27](#_ENREF_27)] and the scale of racial intensity of Ñopo et al. [[28](#_ENREF_28)], an ethnoracial typology will be elaborated and validated for the study, defining in detail the criteria and markers used to define what is considered indigenous and non-indigenous. Given that the study aims to manipulate the perception of obstetricians providing family planning advice, the differences between indigenous and non-indigenous typologies must be credible and salient to the same obstetricians. To validate the ethnoracial types, obstetricians who carry out care work in health establishments in Lima and Callao (see informed consent in Annex 4) will be recruited. The validation of the ethnoracial types will be implemented in two stages:
  - First, a qualitative validation process will be carried out with a sample of 12 obstetricians who will be shown a catalogue of photographs of diverse ethnoracial types interpreted by models contracted for this purpose. This qualitative validation will enable the identification of characteristics relating to phenotype, clothing, personal up-keep and behavior, which distinguish indigenous and non-indigenous types. This process will help focus the recruitment and training of the simulated patients, and will also facilitate data collection and fieldwork supervision.
  - Second, a quantitative validation of ethnoracial types will be carried out with a representative sample of the 1,200 obstetricians working in public health establishments in Lima and Callao [29-32]. The sample size was calculated considering that in order to compare the two ethnoracial types and detect a significant difference of 0.25% on a scale of 0-10, with a power of 90%, and given a standard deviation of 1.58, 311 obstetricians will need to be interviewed. The obstetricians will be shown a catalogue of photographs of diverse ethnoracial types with the aim of measuring the amount of differences perceived between the ethnoracial types played by the simulated patients participating in this study. In addition, the quantitative validation will: i. collect socio-demographic information on the participating obstetricians and ii. Evaluate how frequently the obstetricians attend to patients similar to the ethnoracial types represented in the photograph catalogue.
- The Check List of health care quality indicators will be validated. Once a series of indicators have been selected based on a literature review and current regulations, three experts will be asked to provide their feedback. The recruited experts will be informed of the study objectives and methodology, provided with a copy of the simulated patient script and asked to offer their written opinion about whether they consider that the selected quality indicators adequately represent current regulations and capture all of the key aspects of family planning services.
- Fieldwork staff will be recruited. Individuals will be recruited who share similar characteristics in terms of age, socio-economic and education level, and with the availability, motivation and capacity necessary to participate in the study (for example, she understands the objectives of the study, is capable organising her thoughts, is able to interpret the assigned role, has passed a physical examination coherent with the profile of the assigned script, has no abnormality or illness which could provoke a particular response from the service providers etc.)
- A protocol for collecting, typing and inputting data, including immediate notification via geo-referenced cellular telephones that enable the simulated patient to report her observations by responding to the previously mentioned check list.
- A supervision protocol will be elaborated to include a daily exit interview with the simulated patient which will confirm her location at the moment she made the notification through the geo-referenced cell phone and the itinerary during the visit to the health establishment. The interview will also enable the supervisor to collect payment receipts, prescriptions, referrals and any other documents received during the consultation.

**4.2 Training, script validation and pilot testing**

Based on the team’s previous experience and with support from actors and health professionals, the simulated patients will be carefully trained in order to guarantee the consistency of their performances when interpreting the assigned script and when completing the check list in the observation report.

The training will be carried out in four phases over two weeks. In the first phase, the SPs will undertake role play with the project facilitation team. In the second phase, the SPs will practice the role play with recruited obstetricians. These ‘consultations’ will be filmed and discussed with the participating service providers and the SPs, both individually and in group sessions. Using the validated scripts and the audio-visual material collected during the second phase, the SPs will be trained during the third phase to complete the check list in the service provider performance observation report. Finally, in the fourth phase, health establishments will be visited and the complete script relating to the clinical consultation and the itinerary between ‘stations’ will be practiced. The SPs will begin fieldwork when they are able to demonstrate precision and consistency when interpreting the assigned role and filling in the observation report.

**4.3 Fieldwork**

The fieldwork will be carried out over three and a half weeks during which 18 simulated patients will carry out approximately 39 visits to health establishments. The SPs will be supervised on a daily basis by three supervisors who will also accompany the simulated patients randomly on the programmed visits. The field supervisors will meet weekly with the lead investigators to evaluate the development of fieldwork and the performance of the simulated patients and to present documentation and audio materials collected during supervised visits.

**4.3 Analysis and Reporting**

The databases will be edited, validated and analyzed using the STATA program and an analysis report will be prepared.

**5. Ethical Considerations**

The research protocol will be submitted for approval to the Ethics Committee at the Cayetano Heredia University. Although consent will be obtained from the obstetricians participating in the validation of the ethnoracial types (see Annex 4), the Ethics Committee will be asked to approve the exoneration of the requirement to obtain consent from the health service providers to be evaluated by the simulated patients. This request is based on the fact that this study constitutes an audit of publicly observable behaviour and must consequently avoid bias produced by the Hawthorne effect during observation. It is important to highlight that previous to the fieldwork going ahead, the study will be shared with all relevant agencies of the Ministry of Health, and a formal public presentation of the results will be organized upon completion. The participating obstetricians will not be informed personally about the results of the study in general, nor of the specific observation of which they formed part. As a measure to avoid any risk or adverse effect for the participating service providers, the obstetricians will not be individually identified.

In order to guarantee service provider anonymity and data confidentiality, the instruments used in the study will not contain personal identifiers pertaining to the simulated patients, service providers or the selected health establishments. Both the instruments and the data bases generated will the data collected will be locked with a code that identifies: the simulated patient who made the observation, the supervisor of the simulated patient, the health establishment, the ethnoracial type played by the simulated patient and the number of visits to the health establishment. The health establishment will be assigned a code between 1 and 175, with no relation to the post code, geographic location, or any other variable used in public databases (such as RENAES). This list can only be deciphered using the Census of Establishments in Lima and Callao, which will be locked away by the principal investigator, with access permitted solely to the researchers on the study. The personnel responsible for data entry and quality control will not have access to this document. In no instance will the health service provider of the health establishment be identified by name or surname, or by any form of identification that would enable them to be singled out. Even though the data bases generated by this study will not contain any personal identifiers, only codes, they will be stored on password-protected computers. Likewise, the study source documents (check lists and exit interviews) will also be locked away. Both the source documents and the Census of Establishments in Lima and Callao will be reserved until the study is published, which is expected to occur within one year of its implementation. Finally, it is worth noting that a clause will be included the fieldworkers’ contracts (simulated patients and supervisors previously trained in research ethics) pertaining to confidentiality and data protection.

**6. Timeline**

| **Weekly Activities** | **01** | **02** | **03** | **04** | **05** | **06** | **07** | **08** | **09** | **10** | **11** | **12** | **13** | **14** | **15** | **16** | **17** | **18** | **19** | **20** | **21** | **22** | **23** | **24** | **25** | **26** | **27** | **28** |
| --- | --- | --- | --- | --- | --- | --- | --- | --- | --- | --- | --- | --- | --- | --- | --- | --- | --- | --- | --- | --- | --- | --- | --- | --- | --- | --- | --- | --- |
| Preparation phase |  |  |  |  |  |  |  |  |  |  |  |  |  |  |  |  |  |  |  |  |  |  |  |  |  |  |  |  |
| Training, script validation and pilot testing |  |  |  |  |  |  |  |  |  |  |  |  |  |  |  |  |  |  |  |  |  |  |  |  |  |  |  |  |
| Field work |  |  |  |  |  |  |  |  |  |  |  |  |  |  |  |  |  |  |  |  |  |  |  |  |  |  |  |  |
| Analysis and reporting |  |  |  |  |  |  |  |  |  |  |  |  |  |  |  |  |  |  |  |  |  |  |  |  |  |  |  |  |

**7. References**

1. Benavides, M. and M. Valdivia, *Metas del Milenio y la brecha étnica en el Perú. Versión preliminar.* 2004.

2. Benavides, M., M. Mena, and C. Ponce, *Estado de la niñez indígena en el Perú*, 2010, UNICEF, INEI: Lima.

3. Valdivia, M., *Etnicidad, antecedentes linguísticos y la salud materno infantl en el Perú. Documento de Trabajo*, 2007, Instituto Nacional de Estadística: Lima.

4. Planas, M.E. and N. Valdivia, *Discriminación y Racismo en el Peru: Un estudio sobre modalidades, motivos y lugares de discriminación en Lima y Cuso*, 2009, AES: Lima.

5. Sulmont, D., *Encuesta nacional sobre discriminación social. Informe fial de análisis de resultados*, 2005, DEMUS: Lima.

6. Reyes, E. and N. Valdivia, *Avanzando en la comprensión de las inequidades étnico/raciales en salud: ¿existen prácticas de discriminación hacia la población indígena en los servicios del Estado? Informe final, mimeo*, 2010.

7. Trivelli, C., *Perú*, in *Pueblos Indígenas. pobreza y desarrollo humano en América Latina, 1994-2004*, G. Hall and H. Patrinos, Editors. 2005, Banco Mundial: Washington D.C. p. 219-242.

8. CEPAL, *Atlas sociodemográfico de los pueblos indígenas del Perú* 2011, Santiago de Chile: Naciones Unidas.

9. Smedley, B., A. Stith, and A. Nelson, eds. *Unequal Treatment: Confronting Racial and Ethnic Disparities in Health Care*. Committee on Understanding and Eliminating Racial and Ethnic Disparities in Health Care 2003, The National Academies.

10. Shavers, V.L., et al., *The State of Research on Racial/Ethnic Discrimination in The Receipt of Health Care.* American Journal of Public Health, 2012. 102(5): p. 953-966.

11. Wade, P., *Race and Ethnicity in Latin America*. Critical Studies in Latin America, ed. J. Pearse. 1997, London- Sterling, Virginia: Pluto Press.

12. Krieger, N., *A glossary for social epidemiology.* Journal of Epidemiology and Community Health, 2001(55): p. 693-700.

13. De la Cadena, M., *Indígenas mestizos. Raza y cultura en el Cusco*. 2004 [2000], Lima: Instituto de Estudios Peruanos.

14. Wade, P., *Race, nature and culture. An anthropological perspective* Anthropology, Culture and Society, ed. T. Eriksen, K. Gardner, and J.P. Mitchel. 2002, London- Sterling, Virginia: Pluto Press.

15. Spencer, S., *Race and Ethnicity. Culture, Identity and Representation*. 2006, London and New York: Routledge.

16. Poole, D., *Visión, raza y modernidad. Una economía visual del mundo andino de imágenes.* 2000, Lima: SUR Casa de Estudios del Socialismo.

17. Miles, R., *Apropos the idea of 'race'...again*, in *Theories of race and racism*, J. Solomos and L. Back, Editors. 2000 [1993], Routledge: London. p. 125-143.

18. Twanama, W.A., *Cholear en Lima.* Márgenes. Encuentro y debate, 1992. 5(9): p. 206-240.

19. Nugent, J.G., *El laberinto de la choledad*. 1992, Lima: Fundación Friedrich Ebert.

20. Santos, M., *La cuestión racial: un ajuste de cuentas en tiempos de globalización y postmodernidad.* Debates en Sociología, 2002. 27: p. 133-171.

21. Sulmont, D., *Race, ethnicity, and politics in three Peruvian localities: an analysis of the 2005 CRISE Perceptions Survey in Peru.* Latin American and Caribbean Ethnic Studies, 2011. 6: p. 47-78.

22. León, F., et al., *Providers’ Compliance with the Balanced Counseling Strategy in Guatemala.* Studies in Family Planning, 2005. 36(2): p. 117-126.

23. León, F., et al., *Duración de las sesiones de consejería y cantidad de información relevante que se intercambia: un estudio en clínicas del Perú.* Perspectivas internacionales en Planificación Familiar, 2001. Número especial: p. 2-8.

24. León, F., G. Quiroz, and A. Brazzoduro, *The Reliability of Simulated Clients' Quality-of-Care Ratings.* Studies in Family Planning, 1994. 25(3): p. 184-190.

25. García, P., et al., *Syndromic management of STDs in pharmacies: evaluation and randomised intervention trial.* Sexual Transmitted Infections, 1998: p. S153-158.

26. García, P., et al., *Training pharmacy workers in recognition, management, and prevention of STDs: district-randomized controlled trial.* Bulletin of the World Health Organisation, 2003. 81(11): p. 806-814.

27. Weller, S. and K. Romney, *Systematic Data Collection*. Qualitative Research Methods. Vol. 12. 1988, Newbury Park: Sage Publications Inc.

28. Ñopo, H., J. Saavedra, and M. Torero, *Ethnicity and Earnings in Urban Peru*, in *Discussion Paper Series*2004, IZA: Bonn.

29. Dirección de Salud V Lima Ciudad, *Análisis de la Situación de Salud de la Dirección de Salud V Lima Ciudad 2011*, Oficina de Epidemiología, Editor 2011, Ministerio de Salud: Lima.

30. Dirección Regional de Salud del Calao, *Análisis de la Situación de Salud del Callao 2012*, Oficina de Epidemiología, Editor 2011, Gobierno Regional del Callao: Lima.

31. Dirección de Salud II Lima Sur, *Análisis de la Situación de Salud de la Dirección de Salud II Lima Sur 2011*, Oficina de Epidemiología, Editor 2012, Ministerio de Salud: Lima.

32. Dirección de Salud IV Lima Este, *Análisis de la Situación de Salud de la Dirección de Salud IV Lima Este 2011*, Oficina de Epidemiología, Editor 2011, Ministerio de Salud: Lima.

**Annex 1**

**SCRIPT FOR SIMULATED PATIENT ATTENTING FAMILY PLANNING SERVICES**

She moved to Lima 1 year ago. She is married to a trader. She is 25 years old, with two children (aged 3 years and 10 months). She is not breastfeeding. She has been in a monogamous relationship for 5 years. There is no family violence. She is healthy. Her last cervical smear was 6 months ago. She would like to have more children in the future. She has used condoms (her husband does not like using them). They currently use condoms inconsistently (not always, and incorrectly). She wants to change method. She knows little or nothing about other methods. She does not trust in natural family planning methods. She is afraid of inserting something into her uterus. She does not want the injection (she is scared of needles). She chooses the pill if given the option. She is in the first day of four in her cycle. She refuses a pelvic examination (due to embarrassment). She is an assertive woman (makes eye contact, asks about positive and negative effects of the pill).^[[1]](#footnote-1)^

**Annex 2**

**CHECK LIST**

**[TO BE REPORTED VIA CELULAR PHONE USING EPISURVEYOR]**

| I. Notification upon arrival at the health establishment | |
| --- | --- |
| 1. Insert health establishment code |  |
| 2. Insert date of visit to health establishment |  |
| 3. Insert time of visit to health establishment |  |
| 3.1 Confirm number of visit to health establishment | Ο First  Ο Second  Ο Third |
| 3.2 Confirm type of clothing worn | Ο Type 1: Indigenous  Ο Type 2: Non-indigenous |

| II. Notification at the entrance of the health establishment | |
| --- | --- |
| 4. Insert health establishment code |  |

| III. Notification type of visit | |
| --- | --- |
| 5. Confirm type of visit made | Ο Made appointment for same day  Ο Made appointment for another day (end of questionnaire)  Ο Unable to make appointment (end of questionnaire) |

| IV. Notification of receipt of payment slip | |
| --- | --- |
| 6. Insert health establishment code |  |

| V. Notification upon entering the clinic | |
| --- | --- |
| 7. Insert health establishment code |  |

| VI. Notification upon leaving the clinic | |
| --- | --- |
| 8. Insert health establishment code |  |

| VII. Report on the quality of service in the clinic | | | | |
| --- | --- | --- | --- | --- |
| A. Characteristics of the service provider | | | | |
| 10. Confirm the sex of the service provider who attended you | | Ο Male  Ο Female | | |
| 11. Estimate the age range fo the service provide who attended you | | Ο 20-29 years  Ο 30-39 years  Ο 40-49 years  Ο 50-59 years  Ο Over 60 years | | |
| 12. Between 1 (lowest) and 10 (highest), how White was the service provider who attended you? | |  | | |
| 13. Between 1(lowest) and 10 (highest), how indigenous was the service provider who attended you? | |  | | |
| B. Socio-emotional communication during the consultation | | | | |
| **VARIABLE** | ACTION | | **YES** | **NO** |
| 14 | The service provider appeared tired during the consultation | |  |  |
| 15 | The service provider appeared annoyed during the consultation | |  |  |
| 16 | The service provider seemed rushed during the consultation | |  |  |
| 17 | The service provider greeted you respectfully | |  |  |
| 18 | The service provider ensured there was visual privacy during the consultation | |  |  |
| 19 | The service provider ensured there was audio privacy during the consultation | |  |  |
| 20 | The service provider asked about the motive of the visit | |  |  |
| 21 | The service provider showed kindness during the consultation | |  |  |
| 22 | The service provider paid attention without interruption | |  |  |
| 23 | There were interruptions/distractions during the consultation | |  |  |
| 24 | The service provider treated you with respect | |  |  |
| 25 | The service provider addressed you formally (using ‘Ud’) | |  |  |
| 26 | The service provider criticized or reprimanded you | |  |  |
| 27 | The service provider use clear language | |  |  |
| 28 | The service provider presented the information too quickly to be able to understand | |  |  |
| 29 | The service provider used IEC materials^[[2]](#footnote-2)^ | |  |  |
| 30 | The service provider gave you IEC materials | |  |  |
| 31 | The service provider explained the IEC materials before handing them to you | |  |  |
| 32 | The service provider gave you sufficient time to explain your personal situation | |  |  |
| C. Technical tasks during the consultation | | | | |
|  | C1. GENERAL QUESTIONS | | **YES** | **NO** |
| 33 | The service provider asked your age | |  |  |
| 34 | The service provider asked how many children you have | |  |  |
| 35 | The service provider asked whether you want to have more children | |  |  |
| 36 | The service provider asked which methods you have used or are using currently | |  |  |
| 37 | The service provider asked how you used the methods | |  |  |
| 38 | The service provider asked about your partner’s attitude towards methods you have used or you are using | |  |  |
|  | C2. OPTIONS OF OFFERED METHODS OF ACTION REQUESTED DE METODOS OFRECIDAS O ACCION SOLICITADA | | **YES** | **NO** |
| 39 | The service provider spoke to you about the rhythm method | |  |  |
| 40 | The service provider spoke to you about condoms | |  |  |
| 41 | The service provider spoke to you about vaginal tablets | |  |  |
| 42 | The service provider spoke to you about the pill | |  |  |
| 43 | The service provider spoke to you about the injection | |  |  |
| 44 | The service provider spoke to you about the IUD | |  |  |
| 45 | The service provider spoke to you about tubal ligation | |  |  |
| 46 | The client was asked to select a method | |  |  |
|  | C3. COUNTER-INDICATIONS OF THE PILL | | **YES** | **NO** |
| 47 | The service provider asked you for the date of your last menstruation/suspected pregnancy | |  |  |
| 48 | The service provider asked you is you are currently breastfeeding | |  |  |
| 49 | The service provider asked you whether you have unexplained bleeding | |  |  |
|  | C4. PILL ACTION MECHANISM | | **YES** | **NO** |
| 50 | The service provider explained that it suspends ovulation | |  |  |
| 51 | The service provider explained that it thickens the cervical lining | |  |  |
|  | C5. INSTRUCTIONS FOR USING THE PILL | | **YES** | **NO** |
| 52 | The service provider told you to start using the pill between days 1-5 of your menstruation | |  |  |
| 53 | The service provider told you to take it every day | |  |  |
| 54 | The service provider told you to begin taking the pill again once the packet is finished | |  |  |
| 55 | The service provider indicated that you should take any missed pill immediately | |  |  |
| 56 | The service provider told you that if you forget to take 2 or more pills (1-21) you should wait for bleeding and use a support method. | |  |  |
|  | C6. SECONDARY EFFECTS OF THE PILL | | **YES** | **NO** |
| 57 | The service provider indicated that menstruation could be suspended | |  |  |
| 58 | The service provider indicated that you could suffer from nausea or vomiting | |  |  |
| 59 | The service provider indicated that you could feel dizzy | |  |  |
| 60 | The service provider indicated that you could gain weight | |  |  |
| 61 | The service provider indicated that you could suffer from headaches | |  |  |
|  | C7. PRESCRIPTION AND HANDING OVER OF THE PILL | | **YES** | **NO** |
| 62 | The service provider indicated where to collect the pills during this visitl | |  |  |
| 63. Indicated where you collected the pills | | Ο In the consultation room  Ο In the pharmacy within the establishment  Ο In another place (specify)  Ο No pills were handed over during this consultation | | |
|  | C8. FOLLOW-UP | | **YES** | **NO** |
| 64 | The service provider arranged for a follow-up visit in one month’s time | |  |  |

| VIII. Report of characteristics of the consultation area and waiting room | |
| --- | --- |
| 65. Is the consultation area solely for family planning services? | Ο Yes  Ο No |
| 66. How many people were waiting in the waiting area when you left the consultation? | Ο less than 5  Ο between 5 and 9  Ο between 10 and 19  Ο between 20 and 29  Ο between 30 and 39  Ο more than 40 |
| 67. How many people in the waiting room were using indigenous clothing (such as the *lliclla* or a traditional skirt)? |  |
| 68. Was there a television in the waiting room? | Ο Yes  Ο No |
| 69. Which programmes were being shown on the television while you waited? |  |

| IX. Report on the quality of service of the itinerary between stations | | | | |
| --- | --- | --- | --- | --- |
| 70. Identify station 1 | | Ο Information  Ο Triage in the family planning consultation area  Ο Cashier  Ο Admission  Ο Other (specify) | | |
| 70.1 | The staff greeted you respectfully | | **YES** | **NO** |
| 70.2 | The staff treated you respectfully | |  |  |
| 70.3 | The staff treated you formally | |  |  |
| 70.4 | The staff criticized or reprimanded you | |  |  |
| 70.5 | The service provider used simple language | |  |  |
| 70.6 | The service provider presented the information too quickly for you to be able to understand | |  |  |
| 71. Identify station 2 | | Ο Information  Ο Triage in the family planning consultation area  Ο Cashier  Ο Admission  Ο Other (specify) | | |
| 71.1 | The staff greeted you respectfully | | **YES** | **NO** |
| 71.2 | The staff treated you respectfully | |  |  |
| 71.3 | The staff treated you formally | |  |  |
| 71.4 | The staff criticized or reprimanded you | |  |  |
| 71.5 | The service provider used simple language | |  |  |
| 71.6 | The service provider presented the information too quickly for you to be able to understand | |  |  |
| 72. Identify station 3 | | Ο Information  Ο Triage in the family planning consultation area  Ο Cashier  Ο Admission  Ο Other (specify) | | |
| 72.1 | The staff greeted you respectfully | | **YES** | **NO** |
| 72.2 | The staff treated you respectfully | |  |  |
| 72.3 | The staff treated you formally | |  |  |
| 72.4 | The staff criticized or reprimanded you | |  |  |
| 72.5 | The service provider used simple language | |  |  |
| 72.6 | The service provider presented the information too quickly for you to be able to understand | |  |  |
| 73. Identify station 4 | | Ο Information  Ο Triage in the family planning consultation area  Ο Cashier  Ο Admission  Ο Other (specify) | | |
| 73.1 | The staff greeted you respectfully | | **YES** | **NO** |
| 73.2 | The staff treated you respectfully | |  |  |
| 73.3 | The staff treated you formally | |  |  |
| 73.4 | The staff criticized or reprimanded you | |  |  |
| 73.5 | The service provider used simple language | |  |  |
| 73.6 | The service provider presented the information too quickly for you to be able to understand | |  |  |
| 74. Identisy Station 5 | | Ο Information  Ο Triage in the family planning consultation area  Ο Cashier  Ο Admission  Ο Other (specify) | | |
| 74.1 | The staff greeted you respectfully | | **YES** | **NO** |
| 74.2 | The staff treated you respectfully | |  |  |
| 74.3 | The staff treated you formally | |  |  |
| 74.4 | The staff criticized or reprimanded you | |  |  |
| 74.5 | The service provider used simple language | |  |  |
| 74.6 | The service provider presented the information too quickly for you to be able to understand | |  |  |
| 75. Identidy station 6 | | Ο Information  Ο Triage in the family planning consultation area  Ο Cashier  Ο Admission  Ο Other (specify) | | |
| 75.1 | The staff greeted you respectfully | | **YES** | **NO** |
| 75.2 | The staff treated you respectfully | |  |  |
| 75.3 | The staff treated you formally | |  |  |
| 75.4 | The staff criticized or reprimanded you | |  |  |
| 75.5 | The service provider used simple language | |  |  |
| 75.6 | The service provider presented the information too quickly for you to be able to understand | |  |  |

| X. Report on the cost of services | |
| --- | --- |
| 76. Amount spent for opening a medical record |  |
| 77. Amount spent on the consultation |  |
| 78 Amount spent on the pill |  |
| 79. Total amount spent on other costs |  |

| XI. General observations | |
| --- | --- |
| 80. During the consultation, did anything make you feel uncomfortable or unsure about your role? | Ο Yes  Ο No |
| 81. During the consultation, did you have to improvise responses to unexpected questions from the service provider? | Ο Yes  Ο No |
| 82. Do you think the service provider suspected you at any point? | Ο Yes  Ο No |
| 83. Comment on anything that caught your attention or that you consider worthwhile mentioning. |  |

**Annex 3**

**SIMULATED PATIENT EXIT INTERVIEW**

| I. Visit control data to be registered and reported via mobile phone | |
| --- | --- |
| 1. Supervision date (dd/mm/yy) |  |
| 2. Supervisor code |  |
| 3. Simulated Patient code |  |
| 4. Observation number of the SP |  |
| 5. Type of health establishment visited | Ο Hospital (II-1, II-2, III-1)  Ο Health Center (I-2,I-3,I-4) |
| 6. DISA or DIRESA | Ο Callao  Ο Lima City  Ο South Lima  Ο East Lima |
| 7. Health establishment code (reported by the SP) |  |
| 8. Is the health establishment code reported by the SP correct? | Ο Yes  Ο No (provide the correct code) |
| 9. Date of visit (dd/mm/yy) |  |
| 10. Number of visit (to the same health establishment) | Ο First  Ο Second  Ο Third |
| 11. Type of visit | Ο Made appointment  Ο Made appointment and was attended in the FP clinic  Ο Was attended in the FP clinic  Ο Unable to make appointment of be attended |
| 12. Type of clothing used (reported by the SP) | Ο Type 1: Indigenous  Ο Type 2: Non-indigenous |
| 13. Was this the type of clothing observed by the supervisor? | Ο Yes  Ο No |
| 14. Was the type of clothing used correct? | Ο Yes  Ο No |
| 15. Was the entire notification carried out according to protocol? | Ο Yes  Ο No |
| 16. The following documents supporting the visit are attached | Ο Medical card  Ο Appointment slip  Ο Payment receipt  Ο Pills (specify Nº)  Ο Others (specify) |
| 17. Was the SP accompanied during this visit? | Ο Yes  Ο No |

| II. Semi-structured interview to be recorded with audio recorder |
| --- |
| - What was the station itinerary that you followed during your visit to the health establishment today? Identify the Itinerary of stations form the following flow chart.   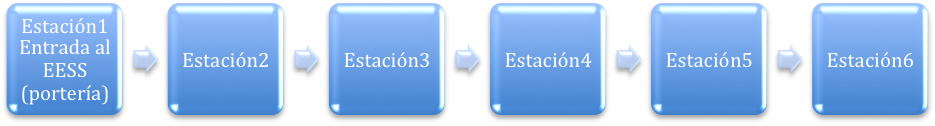   - For each station, indicate:   - Why you approached the station   - Who attended you   - What was the treatment you received   - If you had to pay, how much did you pay?   - Whether you received a document   - Any difficulties encountered - Describe the service provider who attended you during the consultation. What was his/her character like? What was he/she like physically? - What difficulties did you encounter during the consultation in terms of interpreting the role? Indicate the exchanges about each technical task. What difficulties did you have interpreting the role during the consultation?  General questionsOptions of methods offered and action requestedContraindications of the pillHow the pill worksInstructions for using the pillSide-effects of the pillPrescription and handing over the pillFollow-up  - Which questions did the service provider not expect or took him/her by surprise? - Do you think the service provider suspected you at any point? - Did the service provider ask you anything that attracted your attention? - Did anything make you feel uncomfortable or unsure in the role? - Do you have any suggestions to make the role more credible? - Did you encounter any difficulties in notifying and reporting your observations? - Do you have any suggestions for making your job easier? - Orientate and agree with the SP actions to be taken based on discussions during the interview, confirm the next day’s program with the SP and finalize the interview. |

### GENERAL COMMENTS

|  |
| --- |

| Date of supervision (dd/mm/yy) |  |
| --- | --- |
| Name of supervisor |  |
| Supervisor code |  |
| Supervisor’s signature |  |

**Annex Nº4**

INFORMATION SHEET AND CONSENT FORM

**Study into the Quality of Health Services in Indigenous Communities in Peru**

We request your participation as a volunteer in a research study. This sheet explains the study and your role as a participant. Please listen to/ read this information carefully. Take all the time you need. You are a volunteer and may choose not to participate and to leave the study at any point. There will be no penalty should you decide to leave the study. Feel free to ask questions about the research, about issues related to your rights as a volunteer and about any details of the study that are unclear.

TITLE OF THE STUDY

The title of the study is “Quality of Health Services in Indigenous Communities of Peru”

AIM OF THE STUDY

The study is being carried out by the Peruvian University Cayetano Heredia (UPCH) and aims to measure disparities in quality of health care. The activity for which you have been called upon today specifically aims to evaluate the perception of obstetricians towards the diversity of users who attend family planning services. The objective of the research is to validate research instruments and techniques, and generate useful evidence for improving the quality of health care services.

**STUDY PROCEDURES**

Obstetricians will be surveyed, who like you, provide care in Lima and Callao. They will be asked to complete a questionnaire with socio-demographic data, respond to some questions about their care work and evaluate a series of photographs that represent user types. The duration of the interview will be around 20 minutes. Any information that has been recorded or written down during the interview will not be heard or read by anyone other than the team working on the study.

RISKS AND BENEFITS

The study may not benefit you directly, however, the information obtained during the research will contribute to the implementation of studies into the quality of health care, which aim to support the development of public policy with the objective of improving the quality and equality of health service provision in Peru. We will provide you and your network colleagues [indicate Health Network] with an agreeable space and refreshments for carrying out the survey. Once the questionnaires have been completed, we will withdraw and you will be free to use the space to discuss any issues of interest. There are no risks associated with participating in this study.

COSTS AND INCENTIVES

You should not pay anything for participating in this study. Likewise, in addition to refreshments, and the satisfaction of collaborating to better understand the factors that affect the quality of health care in family planning services, you will not receive any economic or other type of incentive.

OPTION TO PARTICIPATE

You have the right to decide whether or not to be interviewed. In addition, at any point during the interview you may ask the interviewer permission to leave and return later on, or ask the interviewer to finalize within a given time.

CONFIDENTIALITY

The information obtained during the interview will be confidential. This means that only the team working on the study will listen to or observe your responses. Your name will never be identified in any report about this study or its results.

CONTACT

If you have any doubts or questions about the study, you may contact Violeta Noa on the following telephone numbers: 382-0318 ó 958802823 or by writing to: [violeta.noa.p@upch.pe](mailto:violeta.noa.p@upch.pe) .

Or the PRINCIPAL INVESTIGATOR:

Name: María Elena Planas

Job title: Associate Professor, Faculty of Public Health and Administration

Address: Av. Honorio Delgado 440

Work telephone number: (511) 382-0318

e-mail: [mariaelena.planas@upch.pe](mailto:mariaelena.planas@upch.pe)

If you have any questions about the ethical aspects of the study, you may contact the President of the IEC, Dr. Fredy Canchihuamán Rivera, by writing to: [duict.cieh@oficinas-upch.pe](mailto:duict.cieh@oficinas-upch.pe), or calling 01-319-0000, annex 2271.

*I have read (I have had read to me) all the information on this form, I have been able to ask questions, I have received any additional information requested, and I give my consent to participating in the study. Likewise, I have been informed that I may have a copy of this form.*

______________________________

Interview date

______________________________

Name of participant

______________________________

Signature of participant

______________________________

Participant code

[Completed by the study team]

**[Study Protocol in the Original Language]**

**Estudio de Calidad de Servicios de Salud**

**en Comunidades Indígenas en el Perú**

**1. Problema y Enfoque conceptual**

Como en otros países latinoamericanos con similares historias de colonización, en el Perú se ha documentado la existencia de brechas etnoraciales en el acceso de salud [[1-3](#_ENREF_1)], pero aún es escasa la evidencia sobre disparidades etnoraciales en la calidad de la atención de salud. Además de estudios cualitativos y encuestas de opinión sobre la percepción de discriminación etnoracial en servicios de salud [[4-6](#_ENREF_4)], no existe aún evidencia cuantitativa sobre cómo el fenotipo o la etnicidad de un paciente puede influenciar la calidad de la prestación de servicios de salud, y en este sentido, cuál es su contribución a las disparidades etnoraciales en la distribución de la salud, enfermedad y bienestar en el país [[2](#_ENREF_2), [7](#_ENREF_7), [8](#_ENREF_8)].

El presente estudio propone evaluar si en el Perú, las características etnoraciales de los pacientes determinan disparidades en la calidad de atención debido a sesgos, prejuicios y estereotipos etnoraciales del prestador de salud; una situación que ha sido reportada extensamente en la literatura internacional [[9](#_ENREF_9), [10](#_ENREF_10)]. Adoptamos el término ‘etnoracial’ dado que, en sociedades con relaciones raciales inequitativas como la peruana, las nociones de etnicidad y raza se traslapan analíticamente y en la práctica [[11-13](#_ENREF_11)]; y definimos raza como una categoría social, no biológica, que hace referencia a diferencias fenotípicas (p.ej., color de piel) producidas como significativas en contextos coloniales para distinguir un ‘nosotros’ europeo de unos ‘otros’ subalternos [[11-17](#_ENREF_11)].

“La cuestión racial en el Perú, señala Twanama (1999), encubre un conglomerado de variables en función de las cuáles las personas se discriminan entre sí … el “choleo” [o la discriminación al indígena es] ejercida sobre alguien considerado racialmente como inferior debido a sus antecedentes indios, pero que es evaluado también en función de aspectos socioeconómicos, educativos-lingüísticos y de la condición de migrante. No es sólo que la plata o un título universitario blanqueen –con cierto límite– sino que la pobreza o lugar de origen también cholean” [[18:212](#_ENREF_18)]. Además de esta multi-dimensionalidad que particulariza la raza al intersecarla con otros ejes de diferenciación social (p. ej., clase, género, sexualidad, etc.) [[4](#_ENREF_4), [18-20](#_ENREF_18)], la discriminación en el Perú es también contextual y situacional. De un lado la localidad o espacio geográfico específico determina qué características y diferencias etnoraciales son significativas, y a quién se reconoce como indígena, mestizo o blanco [[21](#_ENREF_21)], y de otro lado, las características etnoraciales no motivan un trato diferenciado con la misma intensidad y frecuencia en escuelas, servicios de salud, al postular a empleos, etc. [[4](#_ENREF_4)].

Resulta clave en este sentido describir cómo las prácticas discriminatorias se articulan de manera diferenciada en contextos y situaciones diversas, y evaluar al mismo tiempo cuál es la relevancia relativa específica del fenotipo como motivo de discriminación para orientar intervenciones que permitan disminuir las disparidades etnoraciales en el Perú. El presente estudio busca contribuir con este fin, evaluando si existen disparidades etnoraciales en la atención clínica, y si estas disparidades están determinadas por características fenotípicas independientes o articuladas con características culturales.

Con el fin de determinar disparidades etnoraciales en la atención clínica, se propone comparar el grado de consistencia en el desempeño clínico de prestadores de salud con pacientes de tipos etnoraciales diversos. Para ello se evaluará su grado de cumplimiento de estándares de atención clínica relacionados con: a) la duración y costo de la consulta clínica, y b) la calidad de la comunicación prestador-paciente durante la consulta clínica, tanto en su dimensión socioemocional como en relación con tareas técnicas, a decir: el reconocimiento del problema del paciente, el diagnóstico, el manejo (p.ej. consejería, medicación, interconsulta, exámenes auxiliares) y el seguimiento. Adicionalmente se evaluará también la calidad de los itinerarios de pacientes durante su visita al establecimiento de salud, que incluye la interacción con ‘estaciones’ específicas (p.ej., entrada/vigilancia, informes o triaje, módulo de citas, caja, consultorios, etc.).

**2. Objetivos**

***Objetivo general***

Medir disparidades etnoraciales en la calidad de atención de los servicios de salud del Perú.

***Objetivos específicos***

- Validar una tipología etnoracial que identifique los tipos indígena y no-indígena que serán utilizados en el estudio.
- Comparar la duración y el costo de la consulta clínica entre pacientes etnoracialmente diversos.
- Comparar la calidad de la comunicación prestador-paciente durante la consulta clínica entre pacientes etnoracialmente diversos.
- Comparar la calidad del itinerario durante la visita al establecimiento de salud entre pacientes etnoracialmente diversos.

**3. Diseño metodológico**

El presente estudio es un ensayo aleatorizado en el que se comparan las discrepancias en el grado de cumplimiento de estándares de calidad de atención a pacientes etnoracialmente diversos que acuden a los servicios de consejería en planificación familiar del Ministerio de Salud (MINSA). Este servicio clínico, atendido por profesionales de obstetricia, ha sido seleccionado dado que cuenta con protocolos aprobados por el Ministerio de Salud que están bien establecidos –incluso de competencia cultural-, y porque existen instrumentos y guiones disponibles y validados de clientes simulados [[22-24](#_ENREF_22)]. Esta selección se justifica además por la evidencia existente en relación a disparidades etnoraciales en el uso de métodos modernos de planificación familiar [3], una situación cuyas causas podrían estar relacionadas tanto a características de la oferta de servicios (p.ej., discriminación), como a características de la demanda (p.ej. preferencias socioculturales). Este estudio busca específicamente explorar las relaciones causales entre características etnoraciales de las pacientes o usuarias y la calidad de la provisión de la consejería en planificación familiar.

Estudios anteriores han evaluado disparidades en la calidad de la provisión de servicios comparando resultados de salud entre pacientes con diferentes características etnoraciales [4, 27-30]. Sin embargo, una limitación de estos estudios es que no permiten controlar la influencia de la heterogeneidad no observable de los pacientes, que a su vez, hace difícil asegurar que otras características idiosincráticas no observables, más allá de las características etnoraciales, estén afectando los resultados observados. Para superar esta limitación se llevará adelante un experimento inspirado en la metodología usada en economía laboral denominada “audit studies”, los cuales buscan aislar el impacto de la discriminación etnoracial y/o de género. Por ejemplo, en el caso de la evaluación de un postulante a un empleo, se entiende que existe discriminación cuando a dos personas exactamente iguales en características observables como nivel educativo, lugar de residencia, nivel de ingreso, experiencia laboral, etc., con excepción de sus características etnoraciales o de género recibe un trato diferenciado. Siguiendo esta línea de estudios, se usará la técnica de pacientes simulados para manipular experimentalmente la percepción de características etnoraciales de manera de medir disparidades en la prestación de servicios de salud. Adicionalmente, se recogerá información sobre el perfil de los prestadores (p.ej., sexo, edad, profesión, etc.) y de los establecimientos de salud a través de bases de datos de públicas gubernamentales y del reporte de los mismos pacientes simulados.

***3.1 Técnica de Paciente Simulado (PS)***

Los pacientes simulados son actores entrenados que buscan atención médica siguiendo un guión pre-determinado y estandarizado. Estos actores reportan sus observaciones acerca de la atención médica recibida de manera objetiva luego de su visita al establecimiento de salud. La técnica de paciente simulado, que la FASPA/UPCH ha implementado exitosamente en varias oportunidades [[6](#_ENREF_6), [25](#_ENREF_25), [26](#_ENREF_26)], permite reducir los efectos Hawthorne y reducir la variabilidad entre pacientes. Esta técnica tiene además la ventaja, respecto de las entrevistas de salida a pacientes, de lograr observaciones más críticas y confiables, pues al informar a los clientes simulados de los estándares de calidad relacionados con las acciones que observan, su umbral de expectativa frente a su satisfacción es mayor y se estandarizan criterios de calidad, que usualmente varían de manera idiosincrática y cultural, y que pueden discrepar o desconocer lo normado en las guías de atención clínica [[6](#_ENREF_6)].

Para manipular la percepción etnoracial de los pacientes, se han definido dos tipos etnoraciales: Tipo-1: Indígena (fenotipo indígena + vestimenta indígena) y, Tipo-2: No-Indígena (fenotipo mestizo + vestimenta occidental). Se construirán los dos tipos etnoraciales modificando, de manera aleatoria, la apariencia física de los actores, usando maquillaje, peinados y vestimentas diferenciadas. Esto significa que cada paciente simulado buscará ser atendido en los servicios de planificación familiar, interpretando a veces el Tipo-1: Indígena y a veces el Tipo-2: No- Indígena. Esto permitirá evitar los sesgos potenciales que se desprenden de la heterogeneidad no observable entre actores (p.ej., todas las características físicas y otras no físicas como el lenguaje corporal, las actitudes o las maneras de hablar).

***3.2 Tamaño Muestral y selección de la muestra***

Siendo el establecimiento de salud la unidad de análisis del estudio, el tamaño muestral ha sido calculado para la población de establecimientos de salud de Lima Metropolitana y Callao, con excepción de los hospitales especializados y los puestos de salud que no garantizan el número de atenciones en consejería en planificación familiar que permita que los pacientes simulados pasen desapercibidos.

Después de un riguroso sistema de reclutamiento y capacitación (ver acápite 4), 18 pacientes simuladas, mujeres entre 25 y 40 años de edad, visitarán los servicios de planificación familiar de una muestra de 351 establecimientos de salud. El tamaño muestral se ha calculado considerando que 702 observaciones -4 observaciones por establecimiento- permitirán detectar una diferencia significativa del 10% con un poder de 73%, lo que según la literatura internacional serían diferencias aceptables [[10](#_ENREF_10)].

El muestreo será multi-etápico. Se seleccionarán todos los hospitales de Lima y Callao –a excepción de los hospitales especializados- y se elegirá de manera aleatoria una muestra de centros de salud categorizados como I-3 y I-4. Luego se seleccionará de manera aleatoria el orden de visita de los tipos etnoraciales: Orden1, si Tipo-1: Indígena es primero y Tipo-2: No- Indígena es segundo, y Orden2 si Tipo-2: Indígena es primero y Tipo-1: Indígena es segundo. Estos órdenes se asignarán aleatoriamente en bloques completos de tamaño 4, donde 4 corresponde al número de EESS. Finalmente se seleccionará el horario de visita tomando la primera cita disponible como punto de partida.

***3.3 Instrumentos***

Para implementar la técnica de paciente simulado se ha desarrollado los siguientes instrumentos:

1. **un Guión de Paciente Simulado** (ver Anexo 1), que define el motivo de consulta y las características clínicas (p.ej., antecedentes clínicos, etc.), biográficas (p.ej., estado civil, lugar de origen, tipo de empleo, no asegurado, etc.), y comportamentales (p.ej., grado de asertividad) que cada PS deberá interpretar durante la visita al establecimiento de salud y durante la consulta clínica. Se utilizara una adaptación del guión elaborado por León et al. (2008), (ver Anexo 1). Este guión será validado durante la capacitación (ver acápite 4).
2. **una Lista de Chequeo de Indicadores de Calidad de Atención** (ver Anexo 2). A partir de la normativa vigente que establece los estándares de calidad atención de consejería en PF, se han seleccionado una selección de comportamientos clave del proveedor –para evitar los problemas de confiabilidad relacionados la recordación de los numerosos comportamientos-, y preguntas que requieren solo respuestas simples -de sí y no- de manera de evitar juicios subjetivos. Los indicadores seleccionados capturan las siguientes dimensiones de la calidad a ser analizadas durante la consulta clínica: a) la duración y costo de la consulta (ver ítems I-VI y X del instrumento) y, b) la calidad de la comunicación prestador-paciente durante la consulta, tanto los aspectos de la comunicación centrados en tareas técnicas (p.ej., opciones de métodos ofrecidos, contraindicaciones de la píldora, mecanismo de acción de la píldora, seguimiento, etc.), como los aspectos de la comunicación socioemocional (p.ej., los intercambios sociales, la relación emocional positiva o negativa) (ver ítems VII y VIII del instrumento). Además la lista de chequeo permitirá evaluar el flujo y tipo de trato en las diferentes estaciones visitadas en un mismo establecimiento (ver ítem IX del instrumento). Esta lista de chequeo será reportada a través de celulares y validada recurriendo al juicio de expertos (ver acápite 4).
3. **Una Entrevista de Salida a Pacientes Simulados** (ver Anexo 3), que permitirá una supervisión diaria que controlará la calidad del trabajo de campo reportando datos básicos vía celular (ver ítem 1) y grabando en audio una entrevista más cualitativa, que además permitirá describir de manera detallada el itinerario de estaciones en cada visita, identificar las dificultades experimentadas por la paciente simulada durante la visita, recoger sus sugerencias y orientarla de manera oportuna.
   1. ***Modelo de Análisis econométrico***

El objetivo principal del estudio es identificar una relación causal entre las características etnoraciales de un individuo y la calidad en la provisión de servicios de salud recibida. Con este objetivo se diseñó un experimento que envía pacientes simuladas a demandar atención médica, interpretando de manera aleatoria dos tipos etnoraciales diferentes.

La ecuación básica a estimar es la siguiente:

$$Y_{ijt}=\lambda_{t}+\varphi_{j}+\mu_{i}+ \beta{indigenous\_ethnicity race}_{ijt}+X_{ijt}^{'}\delta+\varepsilon_{ijt}$$

donde $Y_{\mathrm{ijt}}$denota el output a medir de interés para la paciente simulada *i* en el hospital/centro de salud *j* el día de la semana *t*. $\lambda_{t}$ denota efectos fijos de tiempo. $\varphi_{j}$ denota efectos fijos de hospital/centro de salud, los cuales controlan por todas las características observables de los hospitales/centro de salud que son invariantes en el tiempo. $\mu_{i}$se denota efectos fijos individuales que capturan la heterogeneidad no observada propia de los pacientes simulados (p.ej., lenguaje corporal, características en el habla y actitudes). $X_{\mathrm{ijt}}$ es un vector exógeno que incluye características observables variables en el tiempo del prestador y del hospital/centro de salud (p.ej., edad, género, y características etnoraciales del prestador). ${indigenous\_ethnicityrace}_{\mathrm{ijt}}$es una variable indicadora que toma el valor de uno cuando la paciente simulada interpreta la etnicidad/raza indígena y toma el valor de cero en caso contrario. $\varepsilon_{\mathrm{ijt}}$denota los errores aleatorio idiosincráticos. Los errores estándares están agrupados a nivel de hospital/centro de salud para permitir correlación entre pacientes dentro de cada hospital/centro de salud. $\beta$ es el parámetro de interés , el cual mide el impacto de las características etnoraciales en la calidad de la atención médica recibida.

**4. Procedimientos**

Para llevar adelante el estudio se llevarán adelante cuatro fases de trabajo que se detallan a continuación.

**4.1 Fase de preparación.**

En esta fase se llevarán adelante las siguiente actividades:

- Se presentará el estudio a instancias pertinentes del Ministerio de Salud y se someterá el protocolo al Comité de Ética de la UPCH.
- Adecuando las técnicas de recolección sistemática propuestas por Weller & Romney [[27](#_ENREF_27)] y la escala de intensidad racial de Ñopo et al. [[28](#_ENREF_28)], se construirá y validará la tipología etnoracial del estudio, definiendo en detalle los criterios y marcadores utilizados para determinar lo que se considerará indígena y no-indígena. Dado que lo que se busca manipular en el estudio es la percepción de las obstetras que atienden servicios de planificación familiar, las diferencias Indígena/No-Indígena tendrán que ser creibles y significativas para las mismas obstetras. Se convocará entonces para la validación de los tipos etnoraciales a obstetras que realizan trabajo asistencial en establecimientos de salud de Lima y Callao (ver consentimiento informado en anexo Nº4). La validación de tipos etnoraciales se implementará en dos momentos:
  - En un primer momento, se implementará una validación cualitativa con una muestra intensional de 12 obstetras, que serán expuestas a un repertorio de fotografías de diversos tipos etnoraciales encarnados por modelos contratadas para tal fin. Esta validación cualitativa permitirá identificar características fenotípicas, de vestido, de arreglo personal y comportamentales que distinguen los tipos indígena y no-indígena, y orientar tanto los procesos de reclutamiento y capacitación de pacientes simuladas como de recolección de información y supervisión del trabajo de campo.
  - En un segundo momento, se implementará una validación cuantitativa de tipos etnoraciales con una muestra representativa de las 1200 obstetras que laboran en los establecimientos de salud del MINSA en Lima y el Callao [29-32]. El tamaño muestral se calculó considerando que para comparar los dos tipos etnoraciales y detectar una diferencia significativa del 0.25% de una escala de 0-10, con un poder de 90%, y dada una desviación estándar de 1.58, se necesitará encuestar por lo menos 311 obstetras. Calculando que 30% de las obstetras que se recluten no roten actualmente por el servicio de planficación familar, se encuestará hasta un máximo de 404 obstetras. Las obstetras serán expuestas a un repertorio de fotografías de diversos tipos etnoraciales con la finalidad de medir la magnitud de las diferencias percibidas entre los tipos etnoraciales encarnados por las mismas pacientes simuladas participantes. Adicionalmente la validación: i. recogerá información sociodemográfica de las obstetras participantes y ii. evaluará con qué frecuencia atienden a usuarias similares a los tipos etnoraciales representados en el repertorio fotográfico.
- Se validará la Lista de Chequeo de indicadores de calidad de atención. Habiéndose seleccionado una serie de indicadores en base a la literatura y la normativa vigente, se recurrirá al juicio de 3 expertos. Los expertos convocados serán informados de los objetivos y metodología del estudio, y emitirán opinión por escrito sobre si consideran que los indicadores de calidad seleccionados traducen adecuadamente la normativa y recogen todos los aspectos clave de la consejería de planificación familiar dado el guión que interpretará la paciente simulada.
- Se reclutarán el personal de campo. Se reclutarán individuos con similares características en cuanto a la edad, nivel socioeconómico y educativo, y con la disponibilidad, motivación y capacidad necesaria para participar en el estudio (p.ej., entiende los objetivos del estudio, tiene capacidad para organizar su pensamiento, tiene capacidad para interpretar el rol asignado, ha pasado una examen físico coherente con el perfil clínico del guión asignado, no tienen ninguna anormalidad o enfermedad que pueda disparar respuestas particulares por parte de los prestadores de salud, etc.).
- Se elaborará un protocolo de recolección, digitación y entrada de datos, que incluye la notificación inmediata a través de celulares geo-referenciados que permitirá al paciente simulado reportar sus observaciones respondiendo a la lista de chequeo antes descrita.
- Se elaborará un protocolo de supervisión que incluye la entrevista de salida diaria con el paciente simulado, la confirmación de su ubicación al momento de la notificación a través del celular geo-referenciado y el recojo de las constancia de pago, prescripciones, órdenes de servicio y cualquier documento recibido durante la consulta clínica y el itinerario durante la visita al establecimiento de salud.

**4.2 Fase de capacitación, validación de guiones y prueba piloto.**

Los clientes simulados serán entrenados cuidadosamente, basándonos en la experiencia previa del trabajo que hemos realizado y apoyándonos con actores y profesionales de salud, y garantizará tanto la consistencia de los desempeños de las PS en su interpretación del guión asignado y en el reporte de la observación llenando la lista de chequeo elaborada para tal fin.

La capacitación durará dos semanas y se implementará en cuatro fases. En la primera fase, las PS realizan juegos de roles con el equipo facilitador del proyecto. En una segunda fase las PS practican el juego de roles con obstetrices invitadas. Estas ‘consultas’ se filman y se discuten con los prestadores invitados y con las PS de manera individual y colectiva. Con guiones validados luego de la segunda fase, y usando el material audiovisual recogido, en la tercera fase se entrena a las PS en el reporte del desempeño del prestador usando la lista de chequeo elaborada para tal fin. Finalmente en la cuarta fase, se visitan establecimientos de salud y se practica el guión completo relacionado con la consulta clínica y el itinerario entre ‘estaciones’. Las PS iniciarán el trabajo de campo cuando hayan logrado precisión y consistencia en la interpretación del rol asignado y en el reporte de observaciones.

**4.3 Fase de trabajo de campo.**

El trabajo de campo se llevará adelante durante 3 meses y medio, durante los cuales 18 pacientes simuladas realizarán un aproximado de 39 visitas a establecimientos de salud, y serán supervisadas en campo diariamente por tres supervisoras que además acompañaran a las pacientes simuladas de manera aleatoria durante las visitas programadas. Las supervisoras de campo se reunirán semanalmente con los investigadores principales para evaluar el desarrollo del trabajo de campo, el desempeño de las pacientes simuladas y presentar la documentación y el material de audio recogido durante la supervisión.

**4.3 Fase de análisis y reporte.**

Se depurarán y validarán y analizarán las bases de datos utilizando el programa STATA y se preparará un reporte analítico.

**5. Consideraciones éticas**

El protocolo de investigación del estudio será sometido para su aprobación al Comité de Ética de la Universidad Peruana Cayetano Heredia. Si bien para la validación de tipos etnoraciales se obtendrá el consentimiento de las obstetras participantes (ver anexo Nº4), se pedirá al Comité de Ética que apruebe la exoneración de la obtención del consentimiento de los prestadores de salud que serán observados por las pacientes simuladas, dado que siendo un estudio de auditoria y de conducta públicamente observada, el estudio exige evitar el sesgo que produciría el efecto Hawthorne en la observación. Es importante señalar que, previo inicio del trabajo de campo, se pondrá el estudio en conocimiento de las instancias pertinentes del Ministerio de Salud y se hará una devolución formal y pública de los resultados terminado el estudio. Las obstetrices participantes no serán informadas individualmente sobre los resultados del estudio en general, ni de la observación particular en la que participarán dado que, como parte de los cuidados que tomará el estudio para evitar cualquier riesgo o efecto adverso potencial para las prestadores de salud participantes, las obstetrices no serán indentificadas individualmente.

Para garantizar el anonimato de los prestadores de salud y la confidencialidad de la información obtenida, los instrumentos utilizados en el estudio no contendrán identificadores personales del paciente simulado, prestador o de los establecimientos de salud seleccionados. Tanto los instrumentos como las bases de datos generadas a partir de los datos recogidos serán rotulados con un código que identifica: al paciente simulado que realice la observación, el número de observación que éste realiza, el supervisor del paciente simulado, el establecimiento de salud, el tipo etnoracial que el paciente simulado ha personificado y el número de visita realizada al mismo establecimiento. Cabe señalar que el código del establecimiento de salud especificará de 1 a 175 los establecimiento seleccionados y no tiene relación con la dirección postal o el número de ubigeo o cualquier variable utilizada en las bases de datos públicas (p.ej., RENAES). Este listado solo podrá ser decodificado a partir del documento Censo de Establecimientos de Lima y Callao, que será guardado en reserva y bajo llave por la investigadora principal y únicamente tendrán acceso a ella los investigadores del estudio. Ni los digitadores ni el personal responsable del control de calidad de los datos tendrán acceso a este documento. En ningún caso se identificará al prestador de salud o al establecimiento de salud con nombre o apellidos o alguna identificación que permita individualizarlos, y aún cuando las bases de datos que se generen en este estudio no contendrán identificadores personales sólo códigos, éstas estarán alojadas en computadoras protegidas por contraseña. De la misma manera, los documentos fuente del estudio (lista de chequeo y entrevista de salida) serán guardados bajo llave. Tanto los documentos fuente del estudio como el Censo de Establecimientos de Lima y Callao serán mantenidos hasta la publicación del estudio, lo que se espera ocurra dentro del primer año de su inicio. Cabe señalar también que se incluirá en el contrato al personal de campo (pacientes simulados y supervisores), previamente capacitados en aspectos éticos de investigación durante la capacitación, una cláusula de confidencialidad y protección de datos.

**6. Cronograma**

| **Actividades por Semana** | **01** | **02** | **03** | **04** | **05** | **06** | **07** | **08** | **09** | **10** | **11** | **12** | **13** | **14** | **15** | **16** | **17** | **18** | **19** | **20** | **21** | **22** | **23** | **24** | **25** | **26** | **27** | **28** |
| --- | --- | --- | --- | --- | --- | --- | --- | --- | --- | --- | --- | --- | --- | --- | --- | --- | --- | --- | --- | --- | --- | --- | --- | --- | --- | --- | --- | --- |
| Fase de Preparación |  |  |  |  |  |  |  |  |  |  |  |  |  |  |  |  |  |  |  |  |  |  |  |  |  |  |  |  |
| Fase de Capacitación, Validación de Guión y Prueba Piloto |  |  |  |  |  |  |  |  |  |  |  |  |  |  |  |  |  |  |  |  |  |  |  |  |  |  |  |  |
| Fase de Trabajo de Campo |  |  |  |  |  |  |  |  |  |  |  |  |  |  |  |  |  |  |  |  |  |  |  |  |  |  |  |  |
| Fase de Análisis y Reporte |  |  |  |  |  |  |  |  |  |  |  |  |  |  |  |  |  |  |  |  |  |  |  |  |  |  |  |  |

**7. Referencias**

1. Benavides, M. and M. Valdivia, *Metas del Milenio y la brecha étnica en el Perú. Versión preliminar.* 2004.

2. Benavides, M., M. Mena, and C. Ponce, *Estado de la niñez indígena en el Perú*, 2010, UNICEF, INEI: Lima.

3. Valdivia, M., *Etnicidad, antecedentes linguísticos y la salud materno infantl en el Perú. Documento de Trabajo*, 2007, Instituto Nacional de Estadística: Lima.

4. Planas, M.E. and N. Valdivia, *Discriminación y Racismo en el Peru: Un estudio sobre modalidades, motivos y lugares de discriminación en Lima y Cuso*, 2009, AES: Lima.

5. Sulmont, D., *Encuesta nacional sobre discriminación social. Informe fial de análisis de resultados*, 2005, DEMUS: Lima.

6. Reyes, E. and N. Valdivia, *Avanzando en la comprensión de las inequidades étnico/raciales en salud: ¿existen prácticas de discriminación hacia la población indígena en los servicios del Estado? Informe final, mimeo*, 2010.

7. Trivelli, C., *Perú*, in *Pueblos Indígenas. pobreza y desarrollo humano en América Latina, 1994-2004*, G. Hall and H. Patrinos, Editors. 2005, Banco Mundial: Washington D.C. p. 219-242.

8. CEPAL, *Atlas sociodemográfico de los pueblos indígenas del Perú* 2011, Santiago de Chile: Naciones Unidas.

9. Smedley, B., A. Stith, and A. Nelson, eds. *Unequal Treatment: Confronting Racial and Ethnic Disparities in Health Care*. Committee on Understanding and Eliminating Racial and Ethnic Disparities in Health Care 2003, The National Academies.

10. Shavers, V.L., et al., *The State of Research on Racial/Ethnic Discrimination in The Receipt of Health Care.* American Journal of Public Health, 2012. 102(5): p. 953-966.

11. Wade, P., *Race and Ethnicity in Latin America*. Critical Studies in Latin America, ed. J. Pearse. 1997, London- Sterling, Virginia: Pluto Press.

12. Krieger, N., *A glossary for social epidemiology.* Journal of Epidemiology and Community Health, 2001(55): p. 693-700.

13. De la Cadena, M., *Indígenas mestizos. Raza y cultura en el Cusco*. 2004 [2000], Lima: Instituto de Estudios Peruanos.

14. Wade, P., *Race, nature and culture. An anthropological perspective* Anthropology, Culture and Society, ed. T. Eriksen, K. Gardner, and J.P. Mitchel. 2002, London- Sterling, Virginia: Pluto Press.

15. Spencer, S., *Race and Ethnicity. Culture, Identity and Representation*. 2006, London and New York: Routledge.

16. Poole, D., *Visión, raza y modernidad. Una economía visual del mundo andino de imágenes.* 2000, Lima: SUR Casa de Estudios del Socialismo.

17. Miles, R., *Apropos the idea of 'race'...again*, in *Theories of race and racism*, J. Solomos and L. Back, Editors. 2000 [1993], Routledge: London. p. 125-143.

18. Twanama, W.A., *Cholear en Lima.* Márgenes. Encuentro y debate, 1992. 5(9): p. 206-240.

19. Nugent, J.G., *El laberinto de la choledad*. 1992, Lima: Fundación Friedrich Ebert.

20. Santos, M., *La cuestión racial: un ajuste de cuentas en tiempos de globalización y postmodernidad.* Debates en Sociología, 2002. 27: p. 133-171.

21. Sulmont, D., *Race, ethnicity, and politics in three Peruvian localities: an analysis of the 2005 CRISE Perceptions Survey in Peru.* Latin American and Caribbean Ethnic Studies, 2011. 6: p. 47-78.

22. León, F., et al., *Providers’ Compliance with the Balanced Counseling Strategy in Guatemala.* Studies in Family Planning, 2005. 36(2): p. 117-126.

23. León, F., et al., *Duración de las sesiones de consejería y cantidad de información relevante que se intercambia: un estudio en clínicas del Perú.* Perspectivas internacionales en Planificación Familiar, 2001. Número especial: p. 2-8.

24. León, F., G. Quiroz, and A. Brazzoduro, *The Reliability of Simulated Clients' Quality-of-Care Ratings.* Studies in Family Planning, 1994. 25(3): p. 184-190.

25. García, P., et al., *Syndromic management of STDs in pharmacies: evaluation and randomised intervention trial.* Sexual Transmitted Infections, 1998: p. S153-158.

26. García, P., et al., *Training pharmacy workers in recognition, management, and prevention of STDs: district-randomized controlled trial.* Bulletin of the World Health Organisation, 2003. 81(11): p. 806-814.

27. Weller, S. and K. Romney, *Systematic Data Collection*. Qualitative Research Methods. Vol. 12. 1988, Newbury Park: Sage Publications Inc.

28. Ñopo, H., J. Saavedra, and M. Torero, *Ethnicity and Earnings in Urban Peru*, in *Discussion Paper Series*2004, IZA: Bonn.

29. Dirección de Salud V Lima Ciudad, *Análisis de la Situación de Salud de la Dirección de Salud V Lima Ciudad 2011*, Oficina de Epidemiología, Editor 2011, Ministerio de Salud: Lima.

30. Dirección Regional de Salud del Calao, *Análisis de la Situación de Salud del Callao 2012*, Oficina de Epidemiología, Editor 2011, Gobierno Regional del Callao: Lima.

31. Dirección de Salud II Lima Sur, *Análisis de la Situación de Salud de la Dirección de Salud II Lima Sur 2011*, Oficina de Epidemiología, Editor 2012, Ministerio de Salud: Lima.

32. Dirección de Salud IV Lima Este, *Análisis de la Situación de Salud de la Dirección de Salud IV Lima Este 2011*, Oficina de Epidemiología, Editor 2011, Ministerio de Salud: Lima.

**Anexo 1**

**GUION DE CLIENTE SIMULADO DE PLANIFICACION FAMILIAR**

Se ha mudado a Lima hace 1 año. Esposa de comerciante. 25 años, dos niños (3 y 10 meses). No está dando de lactar. Está en una relación monógama hace 5 años. No hay violencia familiar. Es saludable. Papanicolaou hace 6 meses. Desea más hijos en el futuro. Ha usado condones (a su esposo le disgusta cada vez que lo usa). Actualmente usa condón de manera inconsistente (no siempre y de manera inadecuada). Quiere cambiar de método. Sabe poco o nada sobre otros métodos. No confía en los métodos de planificación familiar naturales. Tiene miedo de insertar algo en su útero. No quiere inyección (miedo a las agujas). Escogerá las pastillas si le dan la opción. Está en el primer día de los cuatro de su ciclo menstrual. Rechazaría un examen pélvico (por vergüenza). Es una mujer asertiva (mira a los ojos, pregunta sobre consecuencias positivas y negativas de las píldoras).^[[3]](#footnote-3)^

**Anexo 2**

**LISTA DE CHEQUEO**

**[A SER REPORTADA POR CELULARES CON EPISURVEYOR]**

| I. Notificacion a la llegada Al Establecimiento | |
| --- | --- |
| 1. Inserte el código del EESS |  |
| 2. Inserte la fecha de la visita al EESS |  |
| 3. Inserte la hora de la visita al EESS |  |
| 3.1 Confirme el número de visita al EESS | Ο Primera  Ο Segunda  Ο Tercera |
| 3.2 Confirme tipo de vestimenta que lleva puesta | Ο Tipo 1: Indígena  Ο Tipo 2: No-indigena |

| II. Notificacion a la entrada del establecimiento de salud | |
| --- | --- |
| 4. Inserte el código del establecimiento |  |

| III. Notificacion tipo de visita | |
| --- | --- |
| 5. Confirme el tipo de visita que realizó | Ο Saqué cita para hoy.  Ο Saqué cita para otro día (fin del cuestionario)  Ο No pude sacar cita (fin del cuestionario) |

| IV. Notificacion a la recepcion de la boleta de pago | |
| --- | --- |
| 6. Inserte el código del establecimiento |  |

| V. Notificacion a la entrada del consultorio | |
| --- | --- |
| 7. Inserte el código del establecimiento |  |

| VI. Notificacion a la salida del consultorio | |
| --- | --- |
| 8. Inserte el código del establecimiento |  |

| VII. Reporte de la calidad de atencion en consulta | | | | |
| --- | --- | --- | --- | --- |
| A. Características del Prestador | | | | |
| 10. Identifique el sexo del prestador que le dio la consejería | | Ο Hombre  Ο Mujer | | |
| 11. Identifique el rango de edad del prestador que le dio la consejería | | Ο 20-29 años  Ο 30-39 años  Ο 40-49 años  Ο 50-59 años  Ο Mayor de 60 años | | |
| 12. De 1(menos) a 10 (mas) ¿qué tan blanco es el prestador que le dio la consejería? | |  | | |
| 13. De 1(menos) a 10 (mas) ¿qué tan indígena es el prestador que le dio la consejería? | |  | | |
| B. Comunicación Socioemocional durante la consulta | | | | |
| **VARIABLE** | ACCION | | **SI** | **NO** |
| 14 | El prestador se mostró cansado durante la consulta | |  |  |
| 15 | El prestador se mostró molesto durante la consulta | |  |  |
| 16 | El prestador se mostró apurado durante la consulta | |  |  |
| 17 | El prestador lo saludó respetuosamente | |  |  |
| 18 | El prestador aseguró privacidad visual en el consultorio | |  |  |
| 19 | El prestador aseguró privacidad auditiva en el consultorio | |  |  |
| 20 | El prestador le preguntó por el motivo de la visita | |  |  |
| 21 | El prestador mostró agrado porque usted vino a consulta | |  |  |
| 22 | El prestador le prestó toda su atención sin interrupciones | |  |  |
| 23 | Hubo interrupciones, distracciones/distracciones en el consultorio | |  |  |
| 24 | El prestador lo trató con respeto | |  |  |
| 25 | El prestador la trató de usted | |  |  |
| 26 | El prestador lo criticó le llamó la atención o regañó | |  |  |
| 27 | El prestador usó lenguaje fácil de entender | |  |  |
| 28 | El prestador presentó la información demasiado rápida como para que usted pueda entender | |  |  |
| 29 | El prestador usó material de IEC | |  |  |
| 30 | El prestador le entregó material de IEC | |  |  |
| 31 | El prestador le explicó el material de IEC antes de entregárselo | |  |  |
| 32 | El prestador le dió suficiente tiempo para explicar su situación personal | |  |  |
| C. Tareas Técnicas durante la consulta | | | | |
|  | C1. PREGUNTAS GENERALES | | **SI** | **NO** |
| 33 | El prestador le preguntó su edad | |  |  |
| 34 | El prestador le preguntó por su número de hijos | |  |  |
| 35 | El prestador le preguntó por su deseo de tener más hijos | |  |  |
| 36 | El prestador le preguntó por los métodos que ha utilizado o utiliza actualmente | |  |  |
| 37 | El prestador le preguntó en que forma usaba el métodos | |  |  |
| 38 | El prestador le preguntó por actitud de su pareja frene a los métodos que ha utilizado o utiliza actualmente | |  |  |
|  | C2. OPCIONES DE METODOS OFRECIDAS O ACCION SOLICITADA | | **SI** | **NO** |
| 39 | El prestador le habló acerca del Ritmo | |  |  |
| 40 | El prestador le habló acerca de los Condones | |  |  |
| 41 | El prestador le habló acerca las Tabletas vaginales | |  |  |
| 42 | El prestador le habló acerca de la Pildora | |  |  |
| 43 | El prestador le habló acerca de la Ampolla | |  |  |
| 44 | El prestador le habló acerca del DIU | |  |  |
| 45 | El prestador le habló acerca de la Ligadura de trompas | |  |  |
| 46 | Cliente solicitada a elegir un método | |  |  |
|  | C3. CONTRAINDICACIONES DE LA PILDORA | | **SI** | **NO** |
| 47 | El prestador le preguntó su fecha de la última mestruación/sospecha de embarazo | |  |  |
| 48 | El prestador le preguntó si actualmente estaba dando de lactar | |  |  |
| 49 | El prestador le preguntó si tengo sangrados sin explicación | |  |  |
|  | C4. MECANISMO DE ACCION DE LA PILDORA | | **SI** | **NO** |
| 50 | El prestador le dijo que suspende la ovulación | |  |  |
| 51 | El prestador le dijo que espesa el moco cervical | |  |  |
|  | C5. INSTRUCCIONES DE USO DE LA PILDORA | | **SI** | **NO** |
| 52 | El prestador dijo que inicie uso entre 1-5 dia de mestruación | |  |  |
| 53 | El prestador dijo que la tome todos los días | |  |  |
| 54 | El prestador dijo que reinicie la toma al dia siguiente de terminar el paquete | |  |  |
| 55 | El prestador indicó que tome inmediatamente la pastilla olvidada | |  |  |
| 56 | El prestador indicó que si olvido tomar 2 o más pastillas (1-21) que espere el sangrado y use un método de respaldo | |  |  |
|  | C6. EFECTOS SECUNDARIOS DE LA PILDORA | | **SI** | **NO** |
| 57 | El prestador indicó que puede suspenderse su regla | |  |  |
| 58 | El prestador indicó que puede sentir náuseas o vómitos | |  |  |
| 59 | El prestador indicó que puede sentir mareos | |  |  |
| 60 | El prestador indicó que puede aumentar de peso | |  |  |
| 61 | El prestador indicó que puede tener dolores de cabeza | |  |  |
|  | C7. PRESCRIPCION Y ENTREGA DE LA PILDORA | | **SI** | **NO** |
| 62 | El prestador indicó dónde recibirá las píldoras en esta visita | |  |  |
| 63. Indique dónde recibió las píldoras. | | Ο En el mismo consultorio  Ο En la farmacia del establecimiento  Ο En otro lugar (especifique)  Ο No se proporcionó píldoras en esta consulta | | |
|  | C8. SEGUIMIENTO | | **SI** | **NO** |
| 64 | El proveedor acordó cita de seguimieto en un mes | |  |  |

| VIII. Reporte de características del consultorio y su sala de espera | |
| --- | --- |
| 65. ¿El consultorio es exclusivamente de PF? | Ο Sí  Ο No |
| 66. ¿Cuántas personas esperaban en la sala de espera a su salida del consultorio? | Ο menos de 5  Ο entre 5 y 9  Ο entre 10 y 19  Ο entre 20 y 29  Ο entre 30 y 39  Ο más de 40 |
| 67. ¿Cuántas personas en la sala de espera usaban vestimenta indígena (p.ej., lliclla, pollera)? |  |
| 68. ¿Había un televisor en la sala de espera? | Ο Sí  Ο No |
| 69. ¿Qué programa(s) pasaron en la televisión mientras esperaba? |  |

| IX. Reporte de la calidad de atencion del itinerario entre estaciones | | | | |
| --- | --- | --- | --- | --- |
| 70. Identifique la estación 1 | | Ο Información  Ο Triaje en consultorio PF  Ο Caja  Ο Admisión  Ο Otro (especifique) | | |
| 70.1 | El personal lo saludó respetuosamente | | **SI** | **NO** |
| 70.2 | El personal lo trató con respeto | |  |  |
| 70.3 | El personal la trató de usted | |  |  |
| 70.4 | El personal lo criticó le llamó la atención o regañó | |  |  |
| 70.5 | El prestador usó lenguaje fácil de entender | |  |  |
| 70.6 | El prestador presentó la información demasiado rápida como para que usted pueda entender | |  |  |
| 71. Identifique la estación 2 | | Ο Información  Ο Triaje en consultorio PF  Ο Caja  Ο Admisión  Ο Otro (especifique) | | |
| 71.1 | El personal lo saludó respetuosamente | | **SI** | **NO** |
| 71.2 | El personal lo trató con respeto | |  |  |
| 71.3 | El personal la trató de usted | |  |  |
| 71.4 | El personal lo criticó le llamó la atención o regañó | |  |  |
| 71.5 | El prestador usó lenguaje fácil de entender | |  |  |
| 71.6 | El prestador presentó la información demasiado rápida como para que usted pueda entender | |  |  |
| 72. Identifique la estación 3 | | Ο Información  Ο Triaje en consultorio PF  Ο Caja  Ο Admisión  Ο Otro (especifique) | | |
| 72.1 | El personal lo saludó respetuosamente | | **SI** | **NO** |
| 72.2 | El personal lo trató con respeto | |  |  |
| 72.3 | El personal la trató de usted | |  |  |
| 72.4 | El personal lo criticó le llamó la atención o regañó | |  |  |
| 72.5 | El prestador usó lenguaje fácil de entender | |  |  |
| 72.6 | El prestador presentó la información demasiado rápida como para que usted pueda entender | |  |  |
| 73. Identifique la estación 4 | | Ο Información  Ο Triaje en consultorio PF  Ο Caja  Ο Admisión  Ο Otro (especifique) | | |
| 73.1 | El personal lo saludó respetuosamente | | **SI** | **NO** |
| 73.2 | El personal lo trató con respeto | |  |  |
| 73.3 | El personal la trató de usted | |  |  |
| 73.4 | El personal lo criticó le llamó la atención o regañó | |  |  |
| 73.5 | El prestador usó lenguaje fácil de entender | |  |  |
| 73.6 | El prestador presentó la información demasiado rápida como para que usted pueda entender | |  |  |
| 74. Identifique la estación 5 | | Ο Información  Ο Triaje en consultorio PF  Ο Caja  Ο Admisión  Ο Otro (especifique) | | |
| 74.1 | El personal lo saludó respetuosamente | | **SI** | **NO** |
| 74.2 | El personal lo trató con respeto | |  |  |
| 74.3 | El personal la trató de usted | |  |  |
| 74.4 | El personal lo criticó le llamó la atención o regañó | |  |  |
| 74.5 | El prestador usó lenguaje fácil de entender | |  |  |
| 74.6 | El prestador presentó la información demasiado rápida como para que usted pueda entender | |  |  |
| 75. Identifique la estación 6 | | Ο Información  Ο Triaje en consultorio PF  Ο Caja  Ο Admisión  Ο Otro (especifique) | | |
| 75.1 | El personal lo saludó respetuosamente | | **SI** | **NO** |
| 75.2 | El personal lo trató con respeto | |  |  |
| 75.3 | El personal la trató de usted | |  |  |
| 75.4 | El personal lo criticó le llamó la atención o regañó | |  |  |
| 75.5 | El prestador usó lenguaje fácil de entender | |  |  |
| 75.6 | El prestador presentó la información demasiado rápida como para que usted pueda entender | |  |  |

| X. Reporte del costo de la atención | |
| --- | --- |
| 76. Monto gastado por Apertura de Historia |  |
| 77. Monto gastado por la Consulta |  |
| 78. Monto gastado por recibir la Píldora |  |
| 79. Monto total de otros gastos |  |

| XI. Observaciones generales | |
| --- | --- |
| 80. Durante la consulta ¿algo la hizo sentirse incómoda o insegura sobre su rol? | Ο Sí  Ο No |
| 81. Durante la consulta ¿tuvo que improvisar respuestas frente a preguntas inesperadas del prestador | Ο Sí  Ο No |
| 82. ¿Considera que el prestador sospechó en agún momento de usted? | Ο Sí  Ο No |
| 83. Comente lo que le haya llamado la atención o considere relevante mencionar. |  |

**Anexo 3**

**ENTREVISTA DE SALIDA A PACIENTES SIMULADAS**

**[SUPERVISION DIARIA POR VISITA]**

| I. Datos de control de visita a ser registrados y reportados via celular | |
| --- | --- |
| 1. Fecha de la supervisión (dd/mm/aa) |  |
| 2. Código del supervisor |  |
| 3. Código de paciente simulada |  |
| 4. Número de observación de la PS |  |
| 5. Tipo de EESS visitado | Ο Hospital (II-1, II-2, III-1)  Ο Centro de Salud (I-2,I-3,I-4) |
| 6. DISA o DIRESA | Ο Callao  Ο Lima Ciudad  Ο Lima Sur  Ο Lima Este |
| 7. Código del establecimiento visitado (reportado por la PS) |  |
| 8. El código de EESS reportado por la PS es correcto | Ο Si  Ο No (especifique código correcto) |
| 9. Fecha de la visita (dd/mm/aa) |  |
| 10. Número de visita (al mismo EESS) | Ο Primera  Ο Segunda  Ο Tercera |
| 11. Tipo de visita | Ο Sacó cita  Ο Sacó cita y recibió atención en consultorio PF  Ο Recibió atención en consultorio PF  Ο No logró sacar cita ni recibir atención |
| 12. Tipo de vestimenta usada (reportada por PS) | Ο Tipo 1: Indígena  Ο Tipo 2: No-indigena |
| 13. El tipo de vestimenta fue observada por supervisor | Ο Si  Ο No |
| 14. El tipo de vestimenta usada es la correcta | Ο Si  Ο No |
| 15. Logró realizar la notificación completa según protocolo | Ο Si  Ο No |
| 16. Se adjuntan los siguientes documentos sustentatorios de la visita | Ο Carné de atención  Ο Ticket de separación de consulta  Ο Boleta de pago  Ο Píldoras (especifique Nº)  Ο Otros (especifique)  Ο Otros (especifique) |
| 17. Se acompañó a la PS en esta visita | Ο Si  Ο No |

| II. Entrevista semi-estructurada a ser registrada con grabadora de audio |
| --- |
| 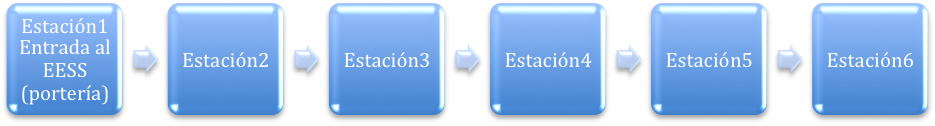   - ¿Cuál fue el itinerario de estaciones que siguió hoy en su visita al EESS? Identifique el itinerario de estaciones en el flujo siguiente. - Retome cada estación e indague:   - Para qué se acercó a la estación.   - Quién la atendió.   - Cuál fue el trato que recibió.   - Si tuvo que pagar, cuánto pago.   - Si recibió un documento.   - Qué dificultades encontró. - Descríbame al(a) prestador(a) que lo atendió durante la consulta ¿Cómo era de carácter? ¿Cómo era físícamente? - ¿Qué dificutades encontró durante la consulta para interpretar su rol? Indague sobre los intercambios en cada una se las tareas técnicas:  Preguntas generalesOpciones de metodos ofrecidas o accion solicitadaContraindicaciones de la pildoraMecanismo de accion de la pildoraInstrucciones de uso de la pildoraEfectos secundarios de la pildoraPrescripcion y entrega de la pildoraSeguimiento  - ¿Qué preguntas del(a) prestador(a) no esperaba o la tomaron por sorpresa? - ¿Considera que el(a) prestador(a) sospechó en algún momento de usted? - ¿Pregunto el(a) prestador(a) algo que le haya llamado la atención? - ¿Algo la hizo sentir incómoda o insegura sobre su rol? - ¿Qué sugeriría para hacer más creible su rol? - ¿Qué dificutades encontró para notificar o reportar sus observaciones? - ¿Qué sugeriría para facilitar su tabajo? - Oriente y acuerde con la PS acciones a ser tomadas en función de lo discutido durante la entrevista, confirme con la PS la programación del día siguiente y finalice la entrevista. |

### COMENTARIOS GENERALES

|  |
| --- |
|  |
|  |
|  |
|  |
|  |

| Fecha de la supervisión (dd/mm/aa) |  |
| --- | --- |
| Nombre de supervisor(a) |  |
| Código del supervisor(a) |  |
| Firma de supervisor(a) |  |

**Anexo Nº4**

DOCUMENTO DE INFORMACIÓN Y FICHA DE CONSENTIMIENTO

**Estudio de Calidad de Servicios de Salud en Comunidades Indígenas del Perú**

Estamos pidiendo su participación como voluntaria en un estudio de investigación. Este formato explica el estudio y su rol como participante. Por favor escuche/ lea esta información cuidadosamente. Tome todo el tiempo que necesite. Usted es voluntaria y puede elegir no participar y dejar el estudio en cualquier momento. No habrá penalidad alguna si usted decide dejar el estudio. Siéntase libre de hacer preguntas acerca de la investigación, de temas relacionados a su derecho como voluntario, y de cualquier detalle acerca del estudio que no esté claro.

TÍTULO DEL ESTUDIO

El título del estudio es “Calidad de Servicios de Salud en Comunidades Indígenas del Perú”.

El PROPÓSITO DEL ESTUDIO

El estudio se encuentra a cargo de la Universidad Peruana Cayetano Heredia (UPCH) y tiene como objetivo medir disparidades en la calidad de atención. La actividad para la que se le ha convocado hoy busca específicamente evaluar las percepción que tienen obstetras sobre la diversidad de usuarias que acuden a los servicios de planificación familiar. La finalidad de la investigación es validar instrumentos y técnicas de investigación y generar evidencia útil para mejorar la calidad de los servicios de salud.

**PROCEDIMIENTOS DEL ESTUDIO**

Se encuestará a obstetras, que como usted, realizan trabajo asistencial en Lima y El Callao. Se les pedirá que llenen un cuestionario con datos sociodemográficos, respondan algunas preguntas sobre su labor asistencial y evalúen una serie de fotografías que representan tipos de usuarias. El tiempo de duración de la entrevista será de aproximadamente 20 minutos. Cualquier información que haya sido grabada o escrita durante la entrevista no será escuchada ni vista por nadie, a excepción del equipo que trabaja en el estudio.

RIESGOS Y BENEFICIOS

El estudio puede que no le beneficie directamente, sin embargo, la información obtenida durante la investigación contribuirá a implementar estudios sobre calidad de atención con el propósito de implementar políticas públicas destinadas a mejorar la calidad y equidad de la provisión de servicios de salud en el Perú. A usted y a sus compañeras de la red [indicar Red de Salud], les facilitaremos un espacio agradable y un refrigerio para realizar el estudio; llenados los cuestionarios, nos retiraremos y ustedes podrán utilizar el espacio para tratar temas que consideren de interés. No se prevén riesgos por participar en este estudio.

COSTOS E INCENTIVOS

Usted no deberá pagar nada por participar en el estudio. Igualmente, más allá del refrigerio que recibirá, y la satisfacción de colaborar con un mejor entendimiento de los factores que afectan la calidad de atención en servicios de planificación familiar, usted no recibirá ningún incentivo económico o de otra índole.

OPCIÓN DE PARTICIPAR

Tiene el derecho de decidir ser o no entrevistada. Además, en cualquier momento de la entrevista, puede solicitar al entrevistador permiso para salir y retornar más tarde o pedir que la entrevista finalice en un tiempo determinado.

CONFIDENCIALIDAD

La información obtenida durante la entrevista será confidencial. Esto significa que sólo se permitirá que el equipo de trabajo del estudio escuche u observe sus respuestas. Su nombre nunca será identificado en ningún reporte de este estudio o de sus resultados.

CONTACTO

Si tiene alguna duda o pregunta sobre el estudio, puede contactar a la Lic. Violeta Noa a los siguientes teléfonos: 382-0318 ó 958802823 o escribiendo a: [violeta.noa.p@upch.pe](mailto:violeta.noa.p@upch.pe) .

o a la INVESTIGADORA PRINCIPAL:

Nombre: María Elena Planas

Cargo: Profesora Asociada, Facultad de Salud Pública y Administración

Dirección: Av. Honorio Delgado 440

Teléfono del trabajo: (511) 382-0318

e-mail: [mariaelena.planas@upch.pe](mailto:mariaelena.planas@upch.pe)

Si usted tiene preguntas sobre los aspectos éticos del estudio puede ponerse en contacto con el presidente del CIE, el Dr. Fredy Canchihuamán Rivera, escribiendo al: [duict.cieh@oficinas-upch.pe](mailto:duict.cieh@oficinas-upch.pe), o llamando al 01-319-0000, anexo 2271.

*He leído (me han leído) toda la información de este formulario, he podido hacer pregunta, he recibido la información adicional solicitada, y he dado mi consentimiento para participar en este estudio. Asimismo, me han informado que puedo tener una copia de este formulario.*

______________________________

Fecha de la entrevista

______________________________

Nombre de la participante

______________________________

Firma de la participante

______________________________

Código del Participante

[llenado por el equipo del estudio]

1. Adapted from León, F., R. Lundgren, et al. (2008). "Provider Selection of Evidence-Based Contraception Guidelines in Service Provision. A Study in India, Peru, and Rwanda." Evaluation & the Health Professions **31**(1): 3-21. [↑](#footnote-ref-1)
2. Information, Education and Communications [↑](#footnote-ref-2)
3. Adaptado de: León, F., R. Lundgren, et al. (2008). "Provider Selection of Evidence-Based Contraception Guidelines in Service Provision.A Study in India, Peru, and Rwanda." Evaluation & the Health Professions **31**(1): 3-21. [↑](#footnote-ref-3)
