# Supplementary material for: Effects of Ethnic Attributes on the Quality of Family Planning Services in Lima, Peru: A Randomized Crossover Trial
Source: PLoS One. 2015 Feb 11;10(2):e0115274. doi: 10.1371/journal.pone.0115274 (PMC4324646; doi:10.1371/journal.pone.0115274)
Supplement: S1 Table — (DOCX) [file pone.0115274.s006.docx]

**Table S1. Validation exercise to assess whether midwives correctly inferred the ethnicity of the SPs.** A representative sample of MoH midwives was asked to assess how indigenous were each of the SPs, with the ethnic assignment of each SP determined at random. The table shows the mean values of perceived indigenousness for every SP enacting an indigenous and mestizo profile, 0 being nothing indigenous and 10 completely indigenous (for further details on the design of the ex-post validation exercise see Planas et al.[1]).

|  | **Mestizo profile** | **Indigenous profile** |  |  |
| --- | --- | --- | --- | --- |
|  | **mean (SD); n=309** | **mean (SD); n=309** | **Differences** | **p value** |
| SP1 | 3.8 (2.47) | 5.0 (2.36) | -1.2 | 0.00 |
| SP2 | 3.8 (2.46) | 5.5 (2.44) | -1.6 | 0.00 |
| SP3 | 4.1 (2.38) | 5.2 (2.32) | -1.0 | 0.00 |
| SP4 | 3.6 (2.48) | 4.7 (2.22) | -1.1 | 0.00 |
| SP5 | 3.4 (2.31) | 5.4 (2.55) | -2.0 | 0.00 |
| SP6 | 3.6 (2.37) | 5.3 (2.45) | -1.7 | 0.00 |
| SP7 | 4.3 (2.61) | 6.5 (2.55) | -2.2 | 0.00 |
| SP8 | 3.6 (2.52) | 5.2 (2.45) | -1.6 | 0.00 |
| SP9 | 3.2 (2.36) | 6.1 (2.56) | -2.8 | 0.00 |
| SP10 | 3.7 (2.49) | 6.4 (2.54) | -2.7 | 0.00 |
| Average Total SPs^a^ | 3.7 (2.46) | 5.5 (2.51) | -1.8 | 0.00 |

^a^The sample size in the Average Total SPs is 3086.

1. Planas ME, García PJ, Bustelo M, Carcamo CP, Ñopo HR, et al. (2014) Using standardized simulated patients to measure ethnic disparities in family planning services in Peru: Study protocol and pre-trial procedures of a crossover randomized trial. Washington, DC: Inter-American Development Bank. Available: http://publications.iadb.org/handle/11319/6387. Accessed 26 March 2014.
