# Supplementary material for: Effects of Ethnic Attributes on the Quality of Family Planning Services in Lima, Peru: A Randomized Crossover Trial
Source: PLoS One. 2015 Feb 11;10(2):e0115274. doi: 10.1371/journal.pone.0115274 (PMC4324646; doi:10.1371/journal.pone.0115274)
Supplement: S2 Table — (DOCX) [file pone.0115274.s007.docx]

**Table S2. Health clinics descriptive statistics reported by SPs.**

|  |  | **Mestizo profile** | **Indigenous profile** |  |  |
| --- | --- | --- | --- | --- | --- |
| **Characteristics** |  | **mean (SD); n=351** | **mean (SD); n=351** | **Differences** | **p value** |
| The office was exclusively for Family Planning |  | 16.2% (36.93%) | 16.2% (36.93%) | 0.0 | 1.00 |
| Number of persons in the waiting room when the SP left the office | [0,4] | 59.3% (49.21%) | 58.1% (49.41%) | 1.1 | 0.75 |
|  | [5,9] | 29.1% (45.47%) | 29.1% (45.47%) | 0.0 | 1.00 |
|  | [10,19] | 10.3% (30.38%) | 10.8% (31.12%) | -0.6 | 0.80 |
|  | [20,29] | 1.4% (11.87%) | 1.7% (12.98%) | -0.3 | 0.76 |
|  | [30,39] | 0.0% (0.00%) | 0.3% (5.34%) | -0.3 | 0.32 |
| Number of people in the waiting room dressed with traditional indigenous clothes (e.g., *lliclla*) |  | 0.4 (1.10) | 0.4 (1.01) | 0.0 | 0.63 |
