# Supplementary material for: Effects of Ethnic Attributes on the Quality of Family Planning Services in Lima, Peru: A Randomized Crossover Trial
Source: PLoS One. 2015 Feb 11;10(2):e0115274. doi: 10.1371/journal.pone.0115274 (PMC4324646; doi:10.1371/journal.pone.0115274)
Supplement: S3 Table — (DOCX) [file pone.0115274.s008.docx]

**Table S3.** **Health provider descriptive statistics reported by SPs.**

|  |  | **Mestizo profile** | **Indigenous profile** |  |  |
| --- | --- | --- | --- | --- | --- |
| **Characteristics** |  | **mean (SD);n=351** | **mean (SD);n=351** | **Differences** | **p value** |
| The health provider is male |  | 5.1% (22.09%) | 4.6% (20.89%) | 0.6 | 0.66 |
| Age of the health provider | [20,29] | 10.0% (30.00%) | 10.5% (30.75%) | -0.6 | 0.78 |
|  | [30,39] | 37.9% (48.58%) | 39.3% (48.91%) | -1.4 | 0.65 |
|  | [40,49] | 35.9% (48.04%) | 36.2% (48.12%) | -0.3 | 0.93 |
|  | [50,59] | 15.1% (35.86%) | 14.0% (34.71%) | 1.1 | 0.65 |
|  | [60,+] | 1.1% (10.63%) | 0.0% (0.00%) | 1.1 | 0.05 |
| How indigenous is the health provider? (1=Less indigenous, 10=More indigenous) | 1 | 11.7% (32.17%) | 10.5% (30.75%) | 1.1 | 0.63 |
|  | 2 | 25.1% (43.40%) | 21.7% (41.25%) | 3.4 | 0.29 |
|  | 3 | 14.5% (35.29%) | 13.4% (34.10%) | 1.1 | 0.67 |
|  | 4 | 10.0% (30.00%) | 12.8% (33.48%) | -2.8 | 0.25 |
|  | 5 | 19.1% (39.36%) | 19.9% (40.01%) | -0.9 | 0.78 |
|  | 6 | 15.7% (36.40%) | 18.5% (38.90%) | -2.8 | 0.31 |
|  | 7 | 3.4% (18.20%) | 2.6% (15.83%) | 0.9 | 0.51 |
|  | 8 | 0.6% (7.54%) | 0.6% (7.54%) | 0.0 | 1.00 |
|  | 9 | 0.0% (0.00%) | 0.0% (0.00%) | 0.0 | 0.00 |
|  | 10 | 0.0% (0.00%) | 0.0% (0.00%) | 0.0 | 0.00 |
