# Supplementary material for: Effects of Ethnic Attributes on the Quality of Family Planning Services in Lima, Peru: A Randomized Crossover Trial
Source: PLoS One. 2015 Feb 11;10(2):e0115274. doi: 10.1371/journal.pone.0115274 (PMC4324646; doi:10.1371/journal.pone.0115274)
Supplement: S4 Table — (DOCX) [file pone.0115274.s009.docx]

**Table S4.** **Differences in the Technical Task Index using multivariate analysis.**

|  | **N** | **β** | **(95% CI)** | **p value** |
| --- | --- | --- | --- | --- |
| **Model (i)** | 702 |  |  |  |
| Indigenous profile |  | 0.74 | (-0.53 to 2.01) | 0.25 |
| Constant |  | 37.49*** | (34.80 to 40.18) | 0.00 |
| **Model (ii)** | 702 |  |  |  |
| Indigenous profile |  | 0.74 | (-0.37 to 1.86) | 0.19 |
| Constant |  | 33.63*** | (29.54 to 37.72) | 0.00 |
| **Model (iii)** | 702 |  |  |  |
| Indigenous profile |  | 0.66 | (-0.53 to 1.84) | 0.28 |
| Constant |  | 33.60*** | (28.02 to 39.18) | 0.00 |
| **Model (iv)** | 694 |  |  |  |
| Indigenous profile |  | 0.64 | (-0.6 to 1.87) | 0.31 |
| Constant |  | 31.69*** | (25.06 to 38.31) | 0.00 |

All models include a control for phase. Model (i) includes patient level fixed effects. Model (ii) includes patient and health clinic level fixed effects. Model (iii) includes patient and health clinic level fixed effects and health provider and clinic characteristics. Model (iv) includes patient, health clinic and day of the week level fixed effects, health provider and clinic characteristics and a control for time of the day of the effective visit (morning or afternoon). Robust SEs. *p<0.1. **p<0.05. ***p<0.01.
